# A Novel and Functionally Diverse Class of Acetylcholine-Gated Ion Channels

**DOI:** 10.1523/JNEUROSCI.1516-22.2022

**Published:** 2023-02-15

**Authors:** Iris Hardege, Julia Morud, Amy Courtney, William R. Schafer

**Affiliations:** ^1^MRC Laboratory of Molecular Biology, Cambridge CB2 0QH, United Kingdom; ^2^Department of Biology, KU Leuven, 3000 Leuven, Belgium

**Keywords:** acetylcholine, *C. elegans*, choline, ion channel, octopamine, tyramine

## Abstract

Fast cholinergic neurotransmission is mediated by acetylcholine-gated ion channels; in particular, excitatory nicotinic acetylcholine receptors play well established roles in virtually all nervous systems. Acetylcholine-gated inhibitory channels have also been identified in some invertebrate phyla, yet their roles in the nervous system are less well understood. We report the existence of multiple new inhibitory ion channels with diverse ligand activation properties in *Caenorhabditis elegans*. We identify three channels, LGC-40, LGC-57, and LGC-58, whose primary ligand is choline rather than acetylcholine, as well as the first evidence of a truly polymodal channel, LGC-39, which is activated by both cholinergic and aminergic ligands. Using our new ligand–receptor pairs we uncover the surprising extent to which single neurons in the hermaphrodite nervous system express both excitatory and inhibitory channels, not only for acetylcholine but also for the other major neurotransmitters. The results presented in this study offer new insight into the potential evolutionary benefit of a vast and diverse repertoire of ligand-gated ion channels to generate complexity in an anatomically compact nervous system.

**SIGNIFICANCE STATEMENT** Here we describe the diversity of cholinergic signaling in the nematode *Caenorhabditis elegans*. We identify and characterize a novel family of ligand-gated ion channels and show that they are preferentially gated by choline rather than acetylcholine and expressed broadly in the nervous system. Interestingly, we also identify one channel gated by chemically diverse ligands including acetylcholine and aminergic ligands. By using our new knowledge of these ligand-gated ion channels, we built a model to predict the synaptic polarity in the *C. elegans* connectome. This model can be used for generating hypotheses on neural circuit function.

## Introduction

Rapid signaling through neuronal networks is essential for producing coordinated behaviors in animals. At the fundamental level, fast neuronal transmission is mediated through neurotransmitter release, resulting in activation of ion channels on the postsynaptic neuron. In the textbook view, based predominately on mammalian systems, there are two major excitatory neurotransmitters, glutamate and acetylcholine (ACh), and two inhibitory neurotransmitters, gamma-aminobutyric acid (GABA) and glycine, which both switch from excitatory to inhibitory signaling during development in mammalian nervous systems (Kirsch, 2006; Tyzio et al., 2007). Glutamate (Glu) acts via a family of tetrameric ligand-gated cation channels, while the remaining neurotransmitters activate the pentameric ligand-gated ion channel (LGICs) superfamily.

Although LGICs are highly conserved across phyla, ligand binding properties and ion selectivity diverge significantly, resulting in a large diversity of mechanisms by which small molecules acting via LGICs can influence the activity in neuronal circuits particularly when channels from invertebrate phyla are considered. For example, insects and nematodes express inhibitory glutamate receptors from the pentameric ligand-gated ion channel superfamily, localized both in neurons and muscles, which are the main targets for achieving the anthelminthic effect of the drug ivermectin (Cully et al., 1994, 1996). Many animals, including insects, nematodes, and mammals, also express LGICs that can be gated by aminergic ligands, including histamine-gated chloride channels (Gisselmann et al., 2002), important for fly visual processing, a number of nematode channels involved in learning and motor control (Pirri et al., 2009; Morud et al., 2021), as well as the excitatory mammalian 5-HT_3_ receptor (Kondo et al., 2013; Lombaert et al., 2018). Even more divergent roles for LGICs have been identified in marine species, where LGICs gated by terpenes and chloroquine function as chemoreceptors in octopus (van Giesen et al., 2020).

Pentameric ligand-gated ion channels can be subdivided into two large clades, the first containing the nicotinic receptors and their paralogues including the serotonin 5-HT_3_ receptors, and the other containing channels more closely related to GABA_A_ receptors (Jones and Sattelle, 2008). The *Caenorhabditis elegans* genome contains several subfamilies that appear to have diversified independently from vertebrate channels during evolution, leading to the existence of several nematode-specific LGIC subfamilies. The *C. elegans* genome contains a number of GABA_A_-like subfamilies, including genes encoding both anion-selective and cation-selective channels (Ranganathan et al., 2000; Yassin et al., 2001; Putrenko et al., 2005; Ringstad et al., 2009; Margie et al., 2013; Jobson et al., 2015). One of these subfamilies consists of genes for both anion-selective and cation-selective monoamine-gated channels, and another of acetylcholine-gated anion channels or channels that are still largely uncharacterized. One member in one of these subgroups, LGC-40 (ligand-gated channel-40), has previously been reported to be a low-affinity serotonin-gated channel also gated by acetylcholine and choline (Ringstad et al., 2009). The properties of the remaining channels in these subgroups, including their ligands, ion selectivity, and expression patterns, are currently unknown.

Here we describe the ligand activation profiles and pharmacological characteristics of five new *C. elegans* LGICs that are all activated by the cholinergic ligands acetylcholine and/or choline. One of these, LGC-39, forms a homomeric anion channel that, in addition to being activated by acetylcholine, appears to be polymodal and is also activated by monoamines. Using publicly available single-cell RNA sequencing (RNAseq) expression data (Taylor et al., 2021) together with our new electrophysiological data, we predict the polarity of synapses in the worm connectome, as well as intracellular localization patterns for uncharacterized LGICs. These results highlight the unexpected functional diversity of cholinergic signaling in the *C. elegans* nervous system.

## Materials and Methods

### *C. elegans* culture.

Unless otherwise specified, *C. elegans* hermaphrodite worms were cultured on NGM (normal growth media) agar plates with OP50 (Stiernagle, 2006). A full list of strains used in this study can be found in Extended Data Table 4-1.

### *Xenopus laevis* oocytes.

Defolliculated *Xenopus laevis* oocytes were obtained from EcoCyte Bioscience and maintained in ND96 solution as follows (in mm): 96 NaCl, 1 MgCl_2_, 5 HEPES, 1.8 CaCl_2_, and 2 KCl, at 16°C for 3–7 d.

### Molecular biology.

Unless otherwise specified, cDNA sequences of *C. elegans* genes were cloned from wild-type N2 worm cDNA [generated by RT-PCR from total worm RNA using Q5 polymerase (New England Biosciences)]. Where multiple isoforms are present, isoform a was used. LGC-39 cDNA was generated by gene synthesis (Thermo Fisher Scientific). For expression in *Xenopus* oocytes, ion channel cDNA sequences were cloned into the KSM vector downstream of a T3 promoter and between *Xenopus* β-globin 5′ and 3′ UTR regions using the HiFi assembly protocol (New England Biosciences). *C. elegans* expression constructs were also generated using the HiFi assembly protocol (New England Biosciences) into the pDESTR4R3II backbone. *C. elegans* genomic DNA (gDNA) sequences were cloned from wild-type N2 gDNA, and expression was verified by the addition of GFP or mKate2 introduced on the same plasmid after an intercistronic splice site (SL2 site). Unless otherwise specified, promoter sequences consist of ∼2 kb of gDNA upstream of the start codon. A full list of primers used in this study can be found in Extended Data Table 4-2.

### CRISPR/Cas9-mediated gene manipulation.

Endogenous tagging of the M3/4 cytosolic loop of *C. elegans* LGIC proteins with GFP was conducted either using the SapTrap protocol (Schwartz and Jorgensen, 2016; Dickinson et al., 2018) for *lgc-39(lj121)*, or by SunyBiotech for *lgc-57(syb3536)*, *lgc-58(syb3562)*, and *lgc-40(syb3594)*.

### RNA synthesis and microinjection.

cRNA was synthesized *in vitro* using the T3 mMessage mMachine transcription kit according to manufacturer protocol to include a 5′ cap (Thermo Fisher Scientific). Before injection RNA was purified using the GeneJET RNA purification kit (Thermo Fisher Scientific). Size-sorted and defolliculated *Xenopus* oocytes (Ecocyte) were placed individually into 96-well plates and injected with 50 nl of 500 ng/µl RNA using the Roboinject system (Multi Channel Systems). When two constructs were coinjected, the total RNA concentration remained at 500 ng/µl, with a 1:1 ratio of the components. Injected oocytes were incubated at 16°C in ND96 solution until the day of recording, typically between 3 and 6 d postinjection.

### Two-electrode voltage-clamp recording and data analysis.

Two-electrode voltage-clamp (TEVC) recordings were conducted using either the Robocyte2 System or a manual setup with an OC-725D amplifier (Multi Channel Systems). Glass electrodes with a resistance ranging from 0.7 to 2 MΩ were pulled on a P1000 Micropipette Puller (Sutter Instrument). Electrodes contained AgCl wires and backfilled with a 1.5 m KCl and 1 m acetic mixture. Unless otherwise stated, oocytes were clamped at –60 mV. Continuous recordings at 500 Hz were made during application of a panel of agonists [ACh, choline, dopamine (DA), tyramine, GABA, glutamate, histamine, 5-HT, betaine, and octopamine]; each agonist was washed for 10 s, unless specified otherwise, followed by a 10–30 s wash (depending on effect size of the first agonist); and data were gathered over at least two occasions, using different batches of oocytes. The typical perfusion rate was 60 µl/s with a bath volume of ∼80 µl, predicting full solution exchange within 1.5–2 s. However, the timing of currents in our traces indicate that the true exchange rate varies. Solution mixing is likely affected by variations in cell and electrode position in each well, which is not controlled or monitored in our automated system. Data were recorded using the RoboCyte2 control software, or WinWCP for manual recordings, and filtered at 10 Hz. Dose–response protocols used 10 s (unless specified otherwise) agonist application pulses with 60 s of washing in ND96 between each dose. Doses for each dose–response curve were adjusted to ensure that both a lower and upper plateau in current were reached. Where this was not possible because of solubility or oocyte health, the highest dose possible was used. Data were gathered over at least two occasions, using different batches of oocytes. Ion selectivity was detected using a voltage-ramp protocol from –80 to +60 mV (20 mV/s) in the presence of the primary agonist in the following three different solutions: ND96, NMDG [*N*-methyl-d-glucamine (Na^+^ free)], and Na gluconate (low Cl^–^) solutions.

### Confocal and cell ID.

Worms were prepared and immobilized with 75 mm NaAzide in M9 and mounted onto 2% agarose in M9 pads. Image stacks were acquired with a 63× water immersion lens on a Leica SP8 or STED or using a 40× oil immersion objective on a Zeiss LSM780. Collapsed z-stack images were generated in Fiji/Image J. Neurons expressing fluorescent reporters were identified by cell shape, position and crossing with the multicolor reference worm NeuroPAL (Yemini et al., 2021).

### Synaptic polarity prediction.

Inhibitory and excitatory chemical synapse predictions for ACh, Glu, and GABA synapses were based on expression levels of appropriate LGICs in postsynaptic cells. Chemical and electrical connectome data were obtained from Wormweb (http://wormweb.org/details.html), LGIC expression data were taken from the Cengen Project using threshold level 4 (Taylor et al., 2021), and ligand and ion selectivity for each channel was based on this work. Previous work and predictions are presented in Extended Data Table 5-1. Binary expression of LGICs for each neurotransmitter in each neural class were based on expression and characterized in the following four groups: only excitatory, only inhibitory, both excitatory and inhibitory, or none. These binary values were used to make the binary expression heatmap. Overall polarity of a synapse was calculated by summing the expression of all inhibitory and all excitatory LGICs for a given neurotransmitter in each cell class. The sum inhibitory was then taken from the sum of excitatory expression, resulting in an overall positive or negative signed expression in each neural class for each neurotransmitter. The ratio of these sums was also calculated to indicate the strength of polarity. It was assumed that each LGIC in each neural class is present equally at all synapses; therefore, each incoming connection could be assigned a polarity based on its receptor expression for that neurotransmitter. The resulting network with polarity was imported into cystoscope (Shannon et al., 2003) for plotting and further analysis. Analysis scripts can be found on GitHub at https://github.com/hiris25/Worm-Connectome-Polarity.

### Expression and cholinergic synapse analysis.

The total number of cholinergic input or output synapses was calculated for each neural class by summing the number of presynapses for each cell that received a synapse from an ACh-producing neuron (incoming synapses) or the total number of postsynapses an ACh-producing neural class makes (outgoing synapses). ACh-producing cells were described by Pereira et al. (2015), including the assumption that all synapses made by an ACh-producing cell also release ACh, even when this cell cotransmits another neurotransmitter. The synapse number for each neuron was taken from the study by White (1986). Expression data were obtained from the study by Taylor et al. (2021) using a threshold of 2. Neural classes were sorted by ACh in- or out- degree, and the expression of each gene was mapped using a heatmap with an upper threshold of 500. For correlation plots, cells that did not express a receptor were removed from the analysis. Correlation between expression level and ACh in- or out- degree was mapped using relplot in the Python seaborn package, and confidence intervals were placed at 68%, corresponding to the SE of the estimate.

### Experimental design and statistical analysis.

For TEVC dose–response data, the peak current for each dose was normalized to the oocyte maximum current using a custom-built Python script (Morud et al., 2021); unless otherwise stated, this was done using *I*/*I*_max_, where *I*_max_ is the largest current generated by the individual oocyte, irrelevant of which dose this occurred in. Since responses can vary between oocytes, *I*_max_ may occur at a particular dose in some oocytes injected with a given channel gene and at a different dose in others, leading to an averaged normalized response that peaks at <1. Normalized data were imported into GraphPad (Prism) and fitted to either a three- or four-parameter nonlinear Hill equation (as stated in figure legends) to obtain the highest degree of fit and calculate the EC_50_. Antagonist dose responses and ion selectivity recordings were conducted using the EC_50_ concentration of the primary agonist. Antagonist dose–response protocols used 10 s agonist plus antagonist windows, with 60 s of ND96 washes between doses. The agonist concentrations remained constant. Antagonist IC_50_ values were calculated using a second custom-built Python script (Morud et al., 2021). Normalized data were imported into GraphPad (Prism) and fitted to a three-parameter nonlinear Hill equation to calculate the IC_50_ value. TEVC ion selectivity data were normalized to maximum current, and the reversal potential difference Δ*E*_Rev_ was calculated using a custom-built Python script (Morud et al., 2021). The resulting individual values or mean, SEM, and *n* values for each construct were imported into GraphPad for further plotting and statistical analysis. Statistically significant differences in Δ*E*_Rev_ were calculated in GraphPad using a two-way ANOVA with Tukey's correction for multiple comparisons. A representative normalized trace for each construct was also generated in GraphPad. *n* values are stated in respective figure legends.

### Data availability.

Python scripts can be found on GitHub at https://github.com/hiris25/TEVC-analysis-scripts and https://github.com/hiris25/Worm-Connectome-Polarity. Aggregated data used for analyzing TEVC data are available on request from author W.R.S. Further information and requests for *C. elegans* strains and plasmids is to be sent to and will be fulfilled by author W.R.S.

## Results

### Deorphanization of uncharacterized LGICs reveals diversity of cholinergic channels

**Figure 1.**
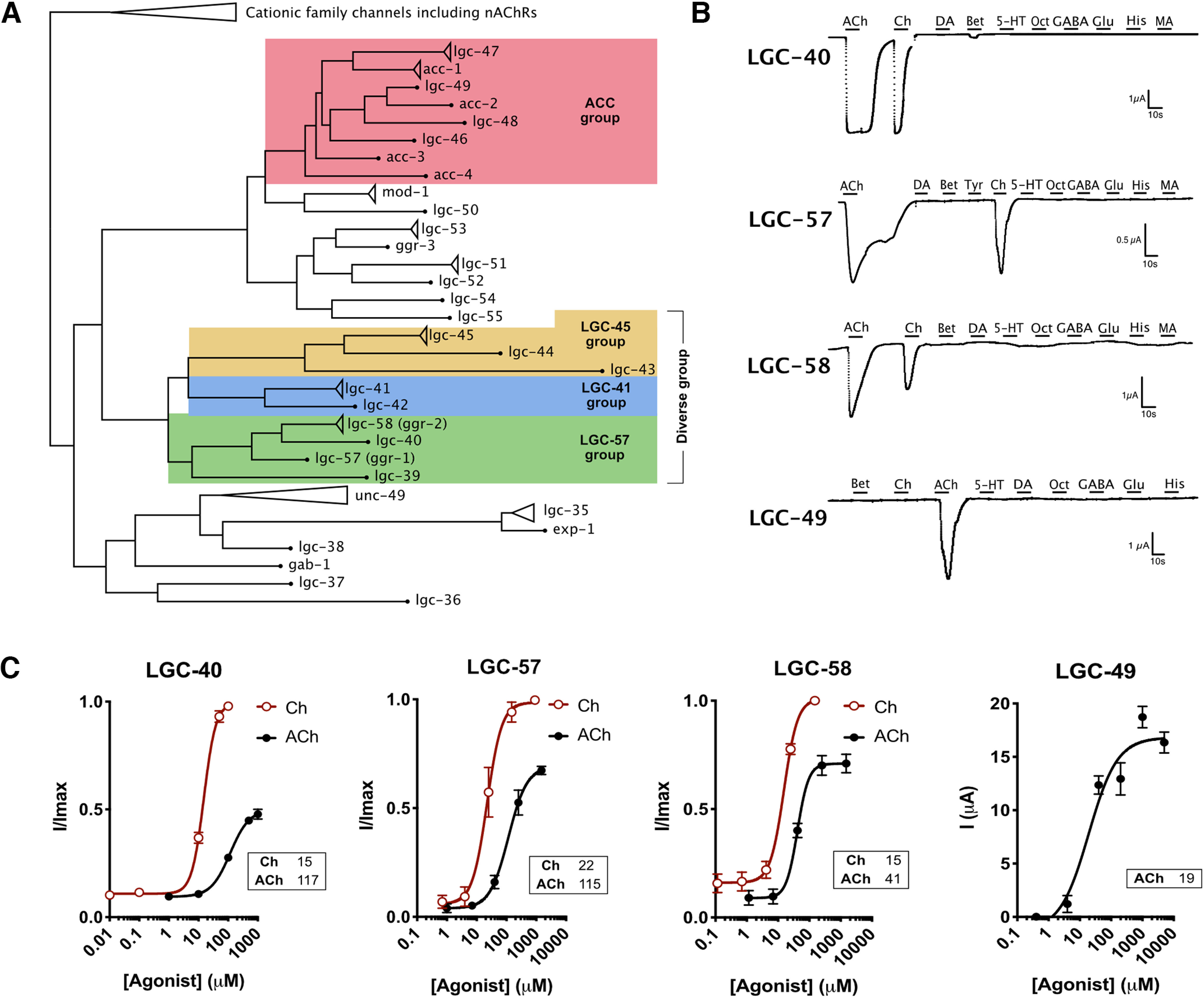
Deorphanization of cholinergic ligand-gated ion channels. ***A***, Simplified phylogenetic tree of subgroups of pentameric ligand-gated ion channels in *C. elegans*; groups of interest are highlighted by color, as follows: ACC group of ACh-gated channels, red; LGC-45 group, yellow; LGC-41 group, blue; and LGC-57 group, green. Triangles represent collapsed isoforms/families, dots represent genes with a single isoform, and branch lengths are not to scale (Extended Data Fig. 1-1, full expanded phylogenetic tree). ***B***, Continuous current traces of *Xenopus* oocytes expressing LGC-40, LGC-57, LGC-58, and LGC-49. Oocytes were perfused for 10 s with a selection of a panel of ligands each at 1 mm: ACh, acetylcholine; Ch, choline; Bet, betaine; dopamine, DA; Tyr, tyramine; 5-HT, serotonin; Oct, octopamine; GABA; His, histamine; MA, melatonin (Extended Data Fig. 1-2, additional traces and ligands). ***C***, Dose–response curves for LGC-40, LGC-57, LGC-58, and LGC-49 in response to their major ligands. Current is normalized by *I*/*I*_max_ for each oocyte; for LGC-49 the current is presented as raw un-normalized current because of difficulties with repeated ligand applications for this specific channel. Error bars represent the SEM of 5–14 oocytes. Curves are fitted with a four-parameter variable slope, and inserts show EC_50_ value in µm for each ligand. Hillslope values: LGC-40: ACh, 1.4; Ch, 2.1; LGC-57: ACh, 1.4; Ch; 1.8; LGC-58: ACh, 2.4, Ch, 2.0 (Extended Data Figs. 1-2, 1-6, additional TEVC traces and alignments; Extended Data Figs. 1-3, 1-4, alignment of the ligand binding loops; Extended Data Fig. 1-5, representative dose–response traces; Extended Data Table 1-1, summary of all channels and ligands tested in this study).

The *C. elegans* genome encodes a diverse superfamily of pentameric LGICs, of which several subfamilies are poorly characterized. Here we investigate the diverse group (for details of group naming, see Jones et al., 2007; Jones and Sattelle, 2008; Hobert, 2013), which consists of three subgroups named after a channel from each group; the LGC-45 group, the LGC-41 group, and the GGR-1 group (here renamed to the “LGC-57 group”; Fig. 1*A*). Collectively the diverse group contains many channels whose activating ligand and function are unknown, which are known as orphan channels. To deorphanize and investigate the properties of these channels, we first expressed each channel gene in *Xenopus* oocytes and tested for current activation during the perfusion of a panel of neurotransmitters. Despite their homology to vertebrate GABA_A_ and glycine receptors (the source of the name GGR-1), we observed no activation of any members of the LGC-57 group (or any diverse group channels) by either GABA or glycine. Instead, we found the three closely related channels of the LGC-57 group—LGC-57 (formerly GGR-1), LGC-58 (formerly GGR-2), and LGC-40—to be specifically gated by choline and acetylcholine (Fig. 1*B*, Extended Data Fig. 1-2*A*). All three channels showed a preference for choline, with EC_50_ values 2.5-fold to threefold lower for choline than acetylcholine (Fig. 1*C*, Extended Data Fig. 1-5). These findings parallel a previous report that LGC-40 forms a choline-gated and acetylcholine-gated channel, although in contrast to that report we did not observe serotonin responses (Ringstad et al., 2009). We did not observe currents in response to any of the tested compounds for the remaining members of the diverse group (LGC-42, LGC-44, LGC-45, or coexpressed as LGC-44/LGC-45), as well as LGC-32, LGC-33, and LGC-34, which, although they did not fall within this group in our phylogenetic analysis (Extended Data Fig. 1-1), have previously been described as part of the diverse group (Hobert, 2013; Extended Data Fig. 1-2*B*,*C*, Extended Data Table 1-1). The lack of agonist-induced currents may be because the channel was poorly expressed, the correct ligand was not tested, or they function only as components of heteromeric complexes. The remaining member of the LGC-57 group, LGC-39, showed unusual activation properties, which will be discussed below.

10.1523/JNEUROSCI.1516-22.2022.f1-1 Figure 1-1 Full phylogenetic tree. Reproduced from the study by Morud et al. (2021). Generated with PHYLIP Neighbor Joining, not to scale. Download Figure 1-1, TIF file.

**Figure 2.**
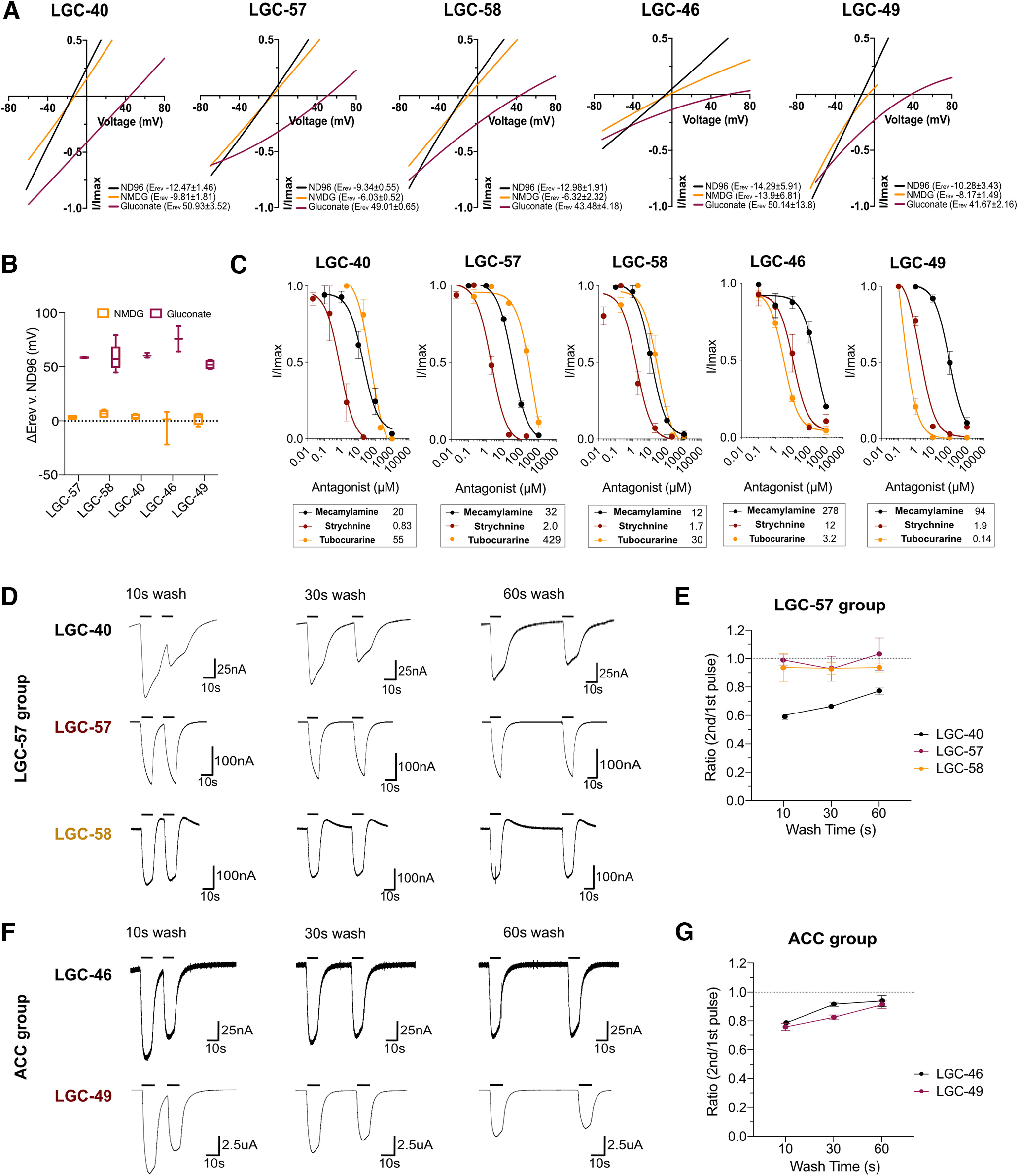
Ion selectivity and antagonistic characterization of cholinergic LGICs. ***A***, Representative current–voltage plots of newly deorphanized channels in ND96, Na^+^ gluconate, or NMDG solutions. Current was normalized by leak current subtraction (in the absence of activating ligand) and the peak current for each oocyte. ***B***, Tukey's box plot of Δ*E*_Rev_ of NMDG and Na^+^ gluconate versus ND96 in oocytes expressing LGC-40, LGC-57, LGC-58, LGC-46, and LGC-49. *E*_Rev_ was calculated in the presence of the primary agonist of each channel and leak subtracted. *N* = 6–11 oocytes. ***C***, Antagonist application in the presence of ligands using mecamylamine, strychnine, and tubocurarine. Current was normalized by *I*/*I*_max_ for each oocyte, curves are fitted with a three-parameter variable slope, error bars represent the SEM of 2–7 oocytes, inserts show IC_50_ values in µm for each antagonist. ***D***, ***F***, Representative traces of oocytes expressing LGC-40, LGC-57, LGC-58, LGC-46, or LGC-49 undergoing repeated agonist application (20 µm choline for LGC-40, LGC-57, and LGC-58; 70 and 20 µm acetylcholine for LGC-46 and LGC-49, respectively) with 10, 30, and 60 s wash intervals in ND96. ***E***, ***G***, Quantification of repeated agonist stimulation, mean current ratio of oocytes at each wash interval is plotted. Error bars represent the SEM. *N* = 5–9 oocytes/condition (Extended Data Fig. 2-1, representative antagonist dose–response traces).

10.1523/JNEUROSCI.1516-22.2022.f1-2 Figure 1-2 Negative traces for still orphan and characterized LGICs. ***A–F***, ***H***, Continuous TEVC traces from oocytes clamped at –60 mV expressing LGICs, exposed to 10 s of a selection of a panel of ligands. Ch, Choline; Bet, betaine; Tyr, tyramine; 5-HT, serotonin; Oct, octopamine; Gly, glycine; His, histamine; MA, melatonin. ***E***, Coexpression of LGC-48 and ACC-4, and not LGC-48 on its own, did not show any agonist-induced current by the ligands tested. ***F***, Continuous TEVC traces from oocytes clamped at –60 mV expressing LGC-47, ACC-1, or a combination, exposed to 10 s of a panel of ligands. Note that the small changes in current seen in the traces are attributed to recording and perfusion artifacts ***G***, ACh-induced dose–response curves for oocytes expressing ACC-1 alone or in combination with LGC-47. Error bars represent the SEM of 7–12 oocytes/construct; insert shows EC_50_ values. ***H***, Continuous TEVC traces from oocytes clamped at –60 mV expressing LGC-39 exposed to 10 s of a panel of ligands. Note that the small changes in current seen in the traces are attributed to recording and perfusion artifacts. Download Figure 1-2, TIF file.

**Figure 3.**
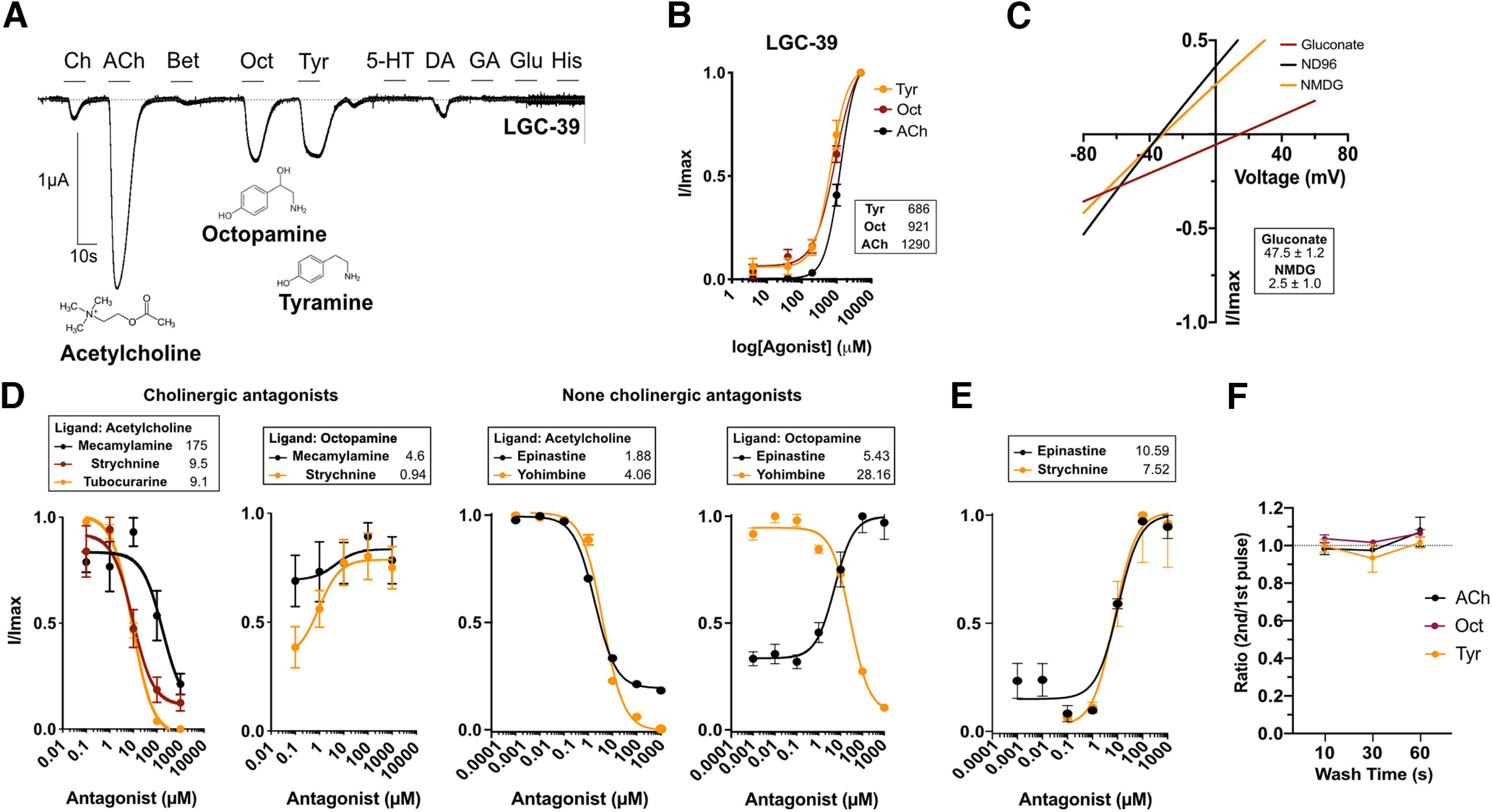
LGC-39 forms a polymodal ligand-gated ion channel. ***A***, Continuous current trace of a *Xenopus* oocyte clamped at −60mV expressing LGC-39, perfused during 10s pulses with a panel of ligands each at 1 mm: Ch, choline; Ach, acetylcholine; Bet, betaine; Tyr, tyramine; 5-HT, serotonin; Oct, octopamine; DA, dopamine; GA, GABA; glutamate, Glu; His, histamine. ***B***, dose–response curve for LGC-39 in response to ACh, Oct, and Tyr. Current is normalized by *I*/*I*_max_ (*I*_max_ at 3 mm agonist) for each oocyte and each compound (dose responses for different agonists could not be done within the same oocytes for technical reasons). Error bars represent the SEM of 8–12 oocytes. Curves are fitted using a four-parameter variable slope; inserts show EC_50_ in µm for each ligand. Hillslope values: Tyr, 1.79; Oct, 1.43; ACh, 1.96. ***C***, Current–voltage relationship during recordings in NMDG, Na^+^ gluconate, or ND96 in oocytes expressing LGC-39. Insert shows Δ*E*_Rev_ versus ND96 in mV ± SEM of 5 oocytes. ***D***, ***E***, Antagonist dose–response curves for LGC-39 expressing oocytes activated by ACh, Oct, or no ligand at a constant dose and varying the antagonist doses (mecamylamine, strychnine, epinastine, and yohimbine). Error bars represent the SEM of 3–7 oocytes. Current is normalized by *I*/*I*_max_, where *I*_max_ is the lowest antagonist dose. Curves are fit with a three-parameter variable slope, inserts show IC_50_ (***D***) or EC_50_ (***E***) values in µm for each ligand. ***F***, Three different agonists do not differ in how they influence the ability for LGC-39 to be stimulated with short time intervals after repeated stimulation (Extended Data Fig. 2-1, representative antagonist dose–response traces).

10.1523/JNEUROSCI.1516-22.2022.f1-3 Figure 1-3 *C. elegans* choline-gated channels show differences in the position of key aromatic residues in the ligand binding domain. ***A***, Alignment of PAR motifs for channels in the diverse and ACC groups. ***B***, Alignment of mouse chrna1 (UniProt ID, P04756) and *C. elegans lgc-40*, *lgc-57*, and *lgc-58*. Red stars highlight key ligand binding residues from mouse *Chrna1*, as described in the study by Bruhova and Auerbach (2017); blue stars highlight the vicinal cysteines of mouse CHRNA1. ***C***, Predicted AlphaFold (Jumper et al., 2021) structures of the ligand binding domains from CHRNA1 (AF-P04756-F1) and LGC-57 (AF-Q09453-F1). Download Figure 1-3, EPS file.

**Figure 4.**
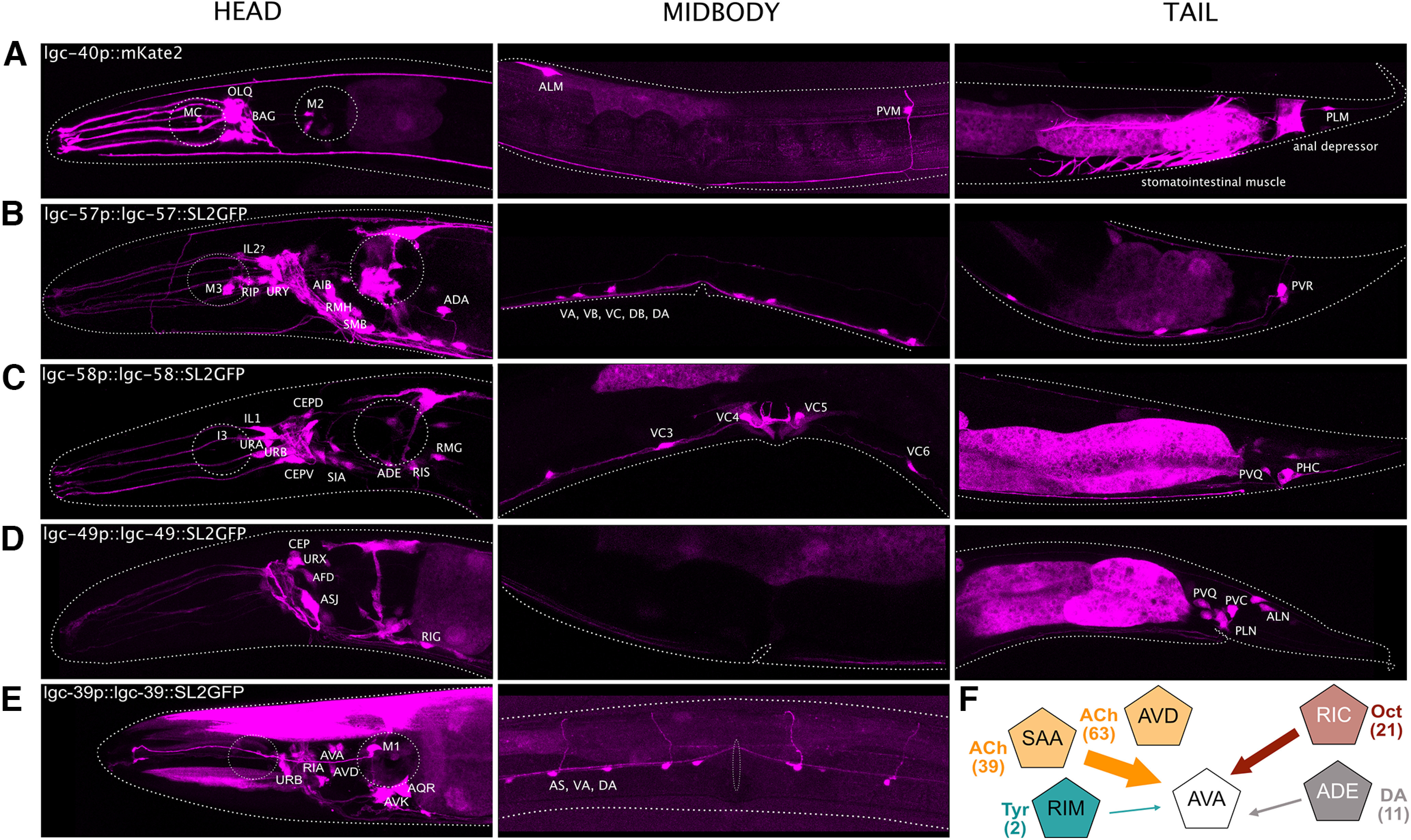
Newly deorphanized LGICs are expressed broadly in the nervous system. ***A–E***, Fluorescent reporters of intercistonically spliced mKate2 or GFP driven under the promoter and/or genomic sequence reveals broad neuronal expression of cholinergic channels with little overlap. *lgc-40* expression was identified in OLQs, BAG, M2, MC, ALM, PVM, and PLM. *lgc-57* expression was identified in M3, RIP, URY, AIB, RMH, SMB, ADA, ventral cord neurons, and PVR. *lgc-58* expression was identified in I3, IL1, CEPs, URA, URB, SIA, ADE, RIS, RMG, VC3-6, PVQ, and PHC. *lgc-49* expression was identified in CEPs, URX, AFD, ASJ, RIG, PVQ, PVC, PLN, and ALN. *lgc-39* expression was identified in URB, RIA, AVA, AVD, M1, AQR, AVK, AS, VA, and DA. ***F***, Schematic depicting a subset of the synaptic connections received by the *lgc-39*-expressing AVA neurons; numbers in brackets show the total number of synapses for each connection (Extended Data Fig. 4-1, cell ID of still orphan LGCs; Extended Data Tables 4-1, strain list, 4-2, primer list, 4-3, expression overlap compared with the CeNGEN dataset).

10.1523/JNEUROSCI.1516-22.2022.f1-4 Figure 1-4 Full alignments of a selection of *C. elegans* pentameric ligand-gated ion channels and mouse *Chrna1*. Generated with CLUSTAL omega, and color applied with CLUSTAL formatting. Download Figure 1-4, TIF file.

**Figure 5.**
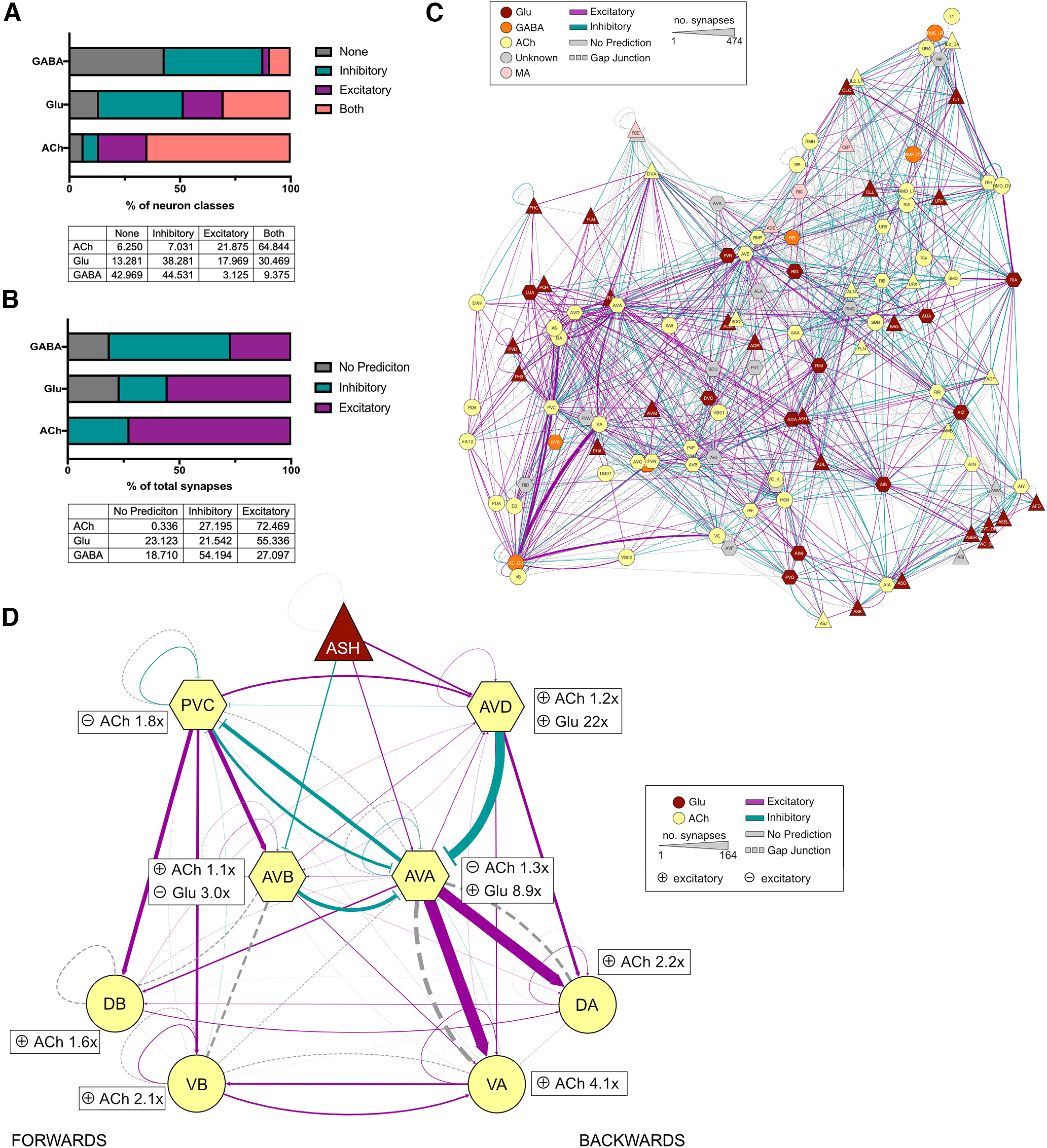
Predicting synapse polarity based on LGIC expression. ***A***, Bar chart and table depicting the percentage of total neuron classes expression inhibitory, excitatory, both or no ionotropic receptors for GABA, Glu, and ACh. ***B***, Bar chart and table depicting the percentage of total synapses for a given neurotransmitter that are predicted to be inhibitory, excitatory, or have no prediction. ***C***, Network diagram depicting the polarity of synaptic connections between neural classes. The connection color shows polarity: teal, inhibitory; pink, excitatory; gray, no prediction. Gap junctions are represented in dashed lines. Line weight represents the number of synapses, and nodes are colored by the major neurotransmitter released by that class. Diagram made with cytoscape using the EntOpt clustering package. ***D***, Network diagram depicting the predicted polarity of the locomotion circuit. The connection color shows polarity: teal, inhibitory; pink, excitatory; gray, no prediction. Inserts next to each neuron node show the fold magnitude of expression of the major receptor type for each neurotransmitter in each neural class (e.g., AVA neurons express 1.3× as many inhibitory ACh receptors than excitatory and 8.9× as many excitatory glutamate receptors than inhibitory). Gap junctions are represented by dashed lines. Line weight represents the number of synapses, and nodes are colored by the major neurotransmitter released by that class. Diagram made with cytoscape (Extended Data Figs. 5-1, binary polarity predictions for each neuronal class, 5-2, expression heatmap of the 3 major neurotransmitters; Extended Data Table 5-1, list of LGC ligands).

10.1523/JNEUROSCI.1516-22.2022.f1-5 Figure 1-5 Representative traces of different doses during dose–response experiments for all characterized channels in this study. Black bars show agonist application time of either 7 or 10 s. Oct, Octopmaine; Tyr, tyramine. Download Figure 1-5, TIF file.

**Figure 6.**
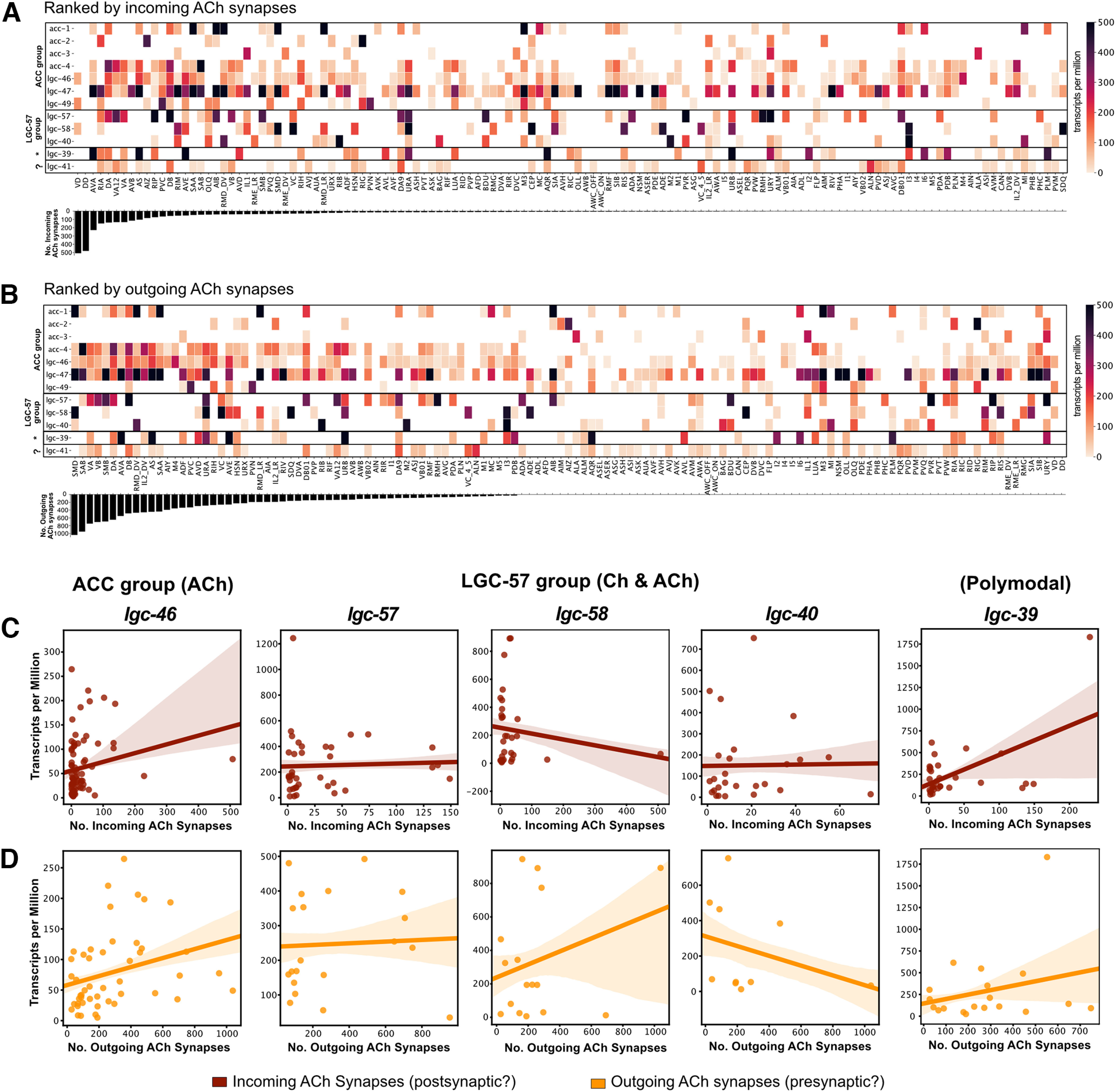
Correlation of cholinergic synapses with expression patterns of cholinergic ion channels. ***A***, ***B***, Heatmaps showing the expression levels of newly deorphanized LGICs in each neural class. Neurons are sorted by the total number of cholinergic synapses they receive (top, Incoming) or make (bottom, Outgoing). ***C***, ***D***, Scatter plots showing correlation between the total number of incoming (red) or outgoing (orange) cholinergic synapses for a given neuronal class and the expression of *lgc-46*, *lgc-57*, *lgc-58*, *lgc-40*, and *lgc-39* (Extended Data Fig. 6-1, correlation graphs of remaining LGCs from this study).

10.1523/JNEUROSCI.1516-22.2022.f1-6 Figure 1-6 Still orphan LGICs coexpressed with RIC-3. ***A***, Representative traces oocytes expressing RIC-3 alone or in combination with LGC-42, LGC-44, LGC-45, LGC-47, and LGC-48. Oocytes were exposed to a 10 s perfusion (indicated by the black bar) of selection of a panel of ligands. ***B***, Quantification of peak current induced after perfusing at 1 mm acetylcholine, choline, and betaine. Download Figure 1-6, TIF file.

10.1523/JNEUROSCI.1516-22.2022.tab1-1 Table 1-1 Overview of LGICs and LGIC combinations screened in this study, including agonists/antagonists and selectivity information. Groups are highlight by color, as follows: ACC group of ACh-gated channels, red; LGC-45 group, yellow; LGC-41 group, blue; LGC-57 group, green. Download Table 1-1, TIF file.

In addition to the diverse group channels, many other *C. elegans* LGICs lack identified ligands. For example, while several members of the ACC (acetylcholine-gated chloride channels) group of LGICs have been shown to form acetylcholine-gated chloride channels, four members of this subfamily (*acc-4*, *lgc-47*, *lgc-48*, and *lgc-49*) had not previously been characterized (Fig. 1*A*, in red). Upon expression in *Xenopus* oocytes, we found that one of these channels, LGC-49, formed a homomeric acetylcholine-gated channel with an EC_50_ of 19 µm (Fig. 1*C*, Extended Data Figs. 1-2*D*, 1-5), similar to the EC_50_ values published for other members of this group (Putrenko et al., 2005; Takayanagi-Kiya et al., 2016). Unlike the members of the LGC-57 group, which showed activation by both acetylcholine and choline, LGC-49 showed no significant activation by choline. This channel further differed from the members in the diverse group by its inability for fast reactivation, which restricted data normalization as per *I*/*I*_max_ (Fig. 1*C*). It should also be noted that the EC_50_ values produced in *Xenopus* oocytes do not necessarily mimic the endogenous *in vivo* EC_50_ of these channels.

10.1523/JNEUROSCI.1516-22.2022.f2-1 Figure 2-1 Representative traces during different antagonist applications. Each channel was exposed to its primary ligand at EC_50_ along with an increasing antagonist concentration. Black bars above the trace show an agonist/antagonist application time of 7 s. Download Figure 2-1, TIF file.

None of the ligands tested here induced currents for ACC-4, LGC-47, or LGC-48 when expressed alone (Extended Data Fig. 1-2*E*,*F*, Extended Data Table 1-1). We note though that a previous study provided evidence that ACC-4 acts as part of a heteromeric complex with ACC-2 (Putrenko et al., 2005). We therefore chose to test the following two combinations of channels from the ACC group: LGC-47 with ACC-1 and LGC-48 with ACC-4 (we did not note any ligand-induced activity for LGC-48/ACC-4; Extended Data Fig. 1-2*E*). However, we did observe a 10-fold right shift in the acetylcholine EC_50_ value between oocytes expressing ACC-1 alone and ACC-1/LGC-47 coexpressing oocytes, with the ACC-1/LGC-47 combination showing a higher EC_50_ value (Extended Data Fig. 1-2*G*). These data suggest that ACC-1 and LGC-47 may form a heteromeric channel; however, further detailed characterization of this combination will be required to validate the existence of a functional heteromer. Given the vast number of possible heteromeric combinations within the ACC group, it may be that these orphan channels are part of more complex channel compositions not tested here. Finally, we attempted to improve expression of the remaining orphan LGICs from the diverse and ACC groups, LGC-42, LGC-44, LGC-45, LGC-47, and LGC-48, by coexpressing these channels with RIC-3, which has previously been shown to enhance the expression of nematode nAChRs in *Xenopus* oocytes (Halevi et al., 2002; Extended Data Fig. 1-6). However, we did not observe any agonist-induced currents in oocytes coexpressing these channels and RIC-3 that were greater than those observed in oocytes expressing RIC-3 alone (Extended Data Fig. 1-6*B*).

We next investigated the ion selectivity of the newly deorphanized channels by carrying out ion substitution experiments in oocytes expressing LGC-40, LGC-57, LGC-58, or LGC-49. For all these channels, we observed significant reversal potential shifts following substitution of the standard high-chloride (NaCl) buffer for a low-chloride (Na gluconate) buffer, but not following substitution with sodium-free (NMDG) solution, indicating selectivity for anions over cations for all the tested channels (Fig. 2*A*,*B*). We also tested the previously deorphanized channel LGC-46 (Takayanagi-Kiya et al., 2016; Liu et al., 2017), which, to date, lacked ion selectivity data. This channel likewise showed reversal shifts characteristic of an anion-selective channel (Fig. 2*A*,*B*). Interestingly, all members of the LGC-57 group possess a PAR (proline-alanine-arginine) motif, located in the M1-2 intracellular loop (Extended Data Fig. 1-3*A*), which has been shown to impart anion selectivity to LGICs (Wotring et al., 2003). Although several uncharacterized members of the ACC group have sequences that diverge from the PAR motif, both LGC-49 and LGC-46 contain the PAR motif sequence (Extended Data Fig. 1-3*A*). Thus, the PAR motif appears to correlate with anion selectivity in both the LGC-57 and ACC groups of nematode acetylcholine-gated LGICs.

### Cholinergic channels display diverse antagonist binding properties

To understand whether there are further functional differences between the channels deorphanized in this study, we exposed each channel to three cholinergic antagonists, mecamylamine, strychnine, and tubocurarine. Strychnine and tubocurarine have been shown to compete with the full agonist for the ligand binding domain, although their binding mechanisms vary between LGICs of different classes (Brams et al., 2011); in contrast, mecamylamine has been shown to interact with the transmembrane regions of mammalian nAChRs (Bondarenko et al., 2014). Indeed, we saw that the antagonistic profile differed significantly between the newly deorphanized channels. For example, within the LGC-57 group the two smallest antagonists, mecamylamine and strychnine, had similar IC_50_ values for LGC-40, LGC-57, and LGC-58 (Fig. 2*C*, Extended Data Fig. 2-1). However, tubocurarine, the largest molecule of the antagonists, displayed an 11-fold shift in IC_50_ for LGC-57 compared with LGC-58 and LGC-40 (Fig. 2*C*, Extended Data Fig. 2-1). Thus, the binding capabilities of tubocurarine on LGC-57 differs substantially from that of its closest family members LGC-58 and LGC-40. Likewise, in the ACC group, LGC-46 and LGC-49 could both be blocked by mecamylamine, strychnine, and tubocurarine (Fig. 2*C*, Extended Data Fig. 2-1). These dissimilarities again highlight the discrete differences between channels from the same subfamily, which may have similar ligand-activation profiles for endogenous ligands. Interestingly, tubocurarine was the most potent blocker for the ACC group channels LGC-46 and LGC-49, whereas this antagonist was the least effective of the channels tested in the LGC-57 group.

We also tested the responses of the channels to repeated stimulation by their primary ligand. We found LGC-40 to be sensitive to repeated stimulation, displaying a significant difference in ratio between the first and second pulse after 10, 30, and 60 s of washing intervals (Fig. 2*D*,*E*). In contrast, all other channels were capable of fast activation intervals as they did not display any decrease in peak amplitude after repeated stimulation (Fig. 2*D–G*). Thus, the mechanism of LGC-40 activation appears to be different from the remainder of the group. However, because of the naturally slow kinetics in *Xenopus* oocytes, it is hard to draw conclusions regarding desensitization or receptor wear down based on these results.

### LGC-39 is a novel polymodal channel activated by cholinergic and aminergic ligands

One channel from the LGC-57 group, LGC-39, showed ligand activation properties distinct from those of the rest of the group. Unlike the other LGC-57 subfamily members, LGC-39 showed relatively little activation by choline (Fig. 3*A*, Extended Data Fig. 1-2*H*). Moreover, while acetylcholine activated LGC-39 strongly (with an EC_50_ of 1290 µm), the most potent ligands for LGC-39 were the monoamines octopamine and tyramine (with EC_50_ values of 921 and 686 µm, respectively; Fig. 3*A*,*B*, Extended Data Fig. 1-5). Although EC_50_ values for LGC-39 were higher than those we observed for other members of the diverse group, EC_50_ values in this range are seen for related channels such as the mammalian GABA_A_ receptors when expressed in *Xenopus* oocytes (Karim et al., 2013). In addition, LGC-39 also displayed small currents in response to dopamine (Fig. 3*A*, Extended Data Fig. 1-2*H*). Both activation by aminergic or cholinergic ligands resulted in Hill slope values >1, suggesting that each ligand binds in a positive cooperative manner on the channel (Cattoni et al., 2015). Like other members of the LGC-57 family, LGC-39 contains the PAR sequence (Extended Data Fig. 1-3*A*), and LGC-39-expressing oocytes showed reversal potential shifts in response to chloride but not sodium substitution (Fig. 3*C*). Thus, *lgc-39* appears to encode a homomeric anionic and polymodal channel, capable of being activated by both aminergic and cholinergic neurotransmitters (Fig. 3*A–C*).

We tested the effects of cholinergic and noncholinergic antagonists on LGC-39 currents evoked by different activating ligands. In the presence of acetylcholine, LGC-39 could be blocked by the cholinergic antagonists mecamylamine, strychnine, and tubocurarine (Fig. 3*D*). In contrast, the octopamine response could not be blocked by mecamylamine or strychnine. Surprisingly, strychnine, without the presence of an activating ligand, acted as a partial agonist, since it induced a small current with an EC_50_ of 7.5 µm (Fig. 3*E*). The noncholinergic blocker, epinastine, a selective octopaminergic blocker (Packham et al., 2010), and yohimbine, an α2 adrenergic blocker, both blocked acetylcholine-induced currents with IC_50_ values of 1 and 4 µm, respectively (Fig. 3*D*); however, only yohimbine blocked octopamine-induced currents with an IC_50_ of 28 µm. Interestingly, epinastine also acted as an agonist both in the presence and absence of octopamine with an EC_50_ of 10 µm (Fig. 3*E*). To further separate the functionality of the ligands, we also investigated whether repeated activation by the different ligands influenced the ability for reactivation of LGC-39 differently (Fig. 3*F*). No difference was seen for any wash interval between the ligands, which could suggest that all ligands occupy the binding site in a similar time frame or that the recovery time for the channel is independent of the activating ligand.

### Cholinergic channels show broad and varied expression in the *C. elegans* nervous system

To gain insight into the roles of cholinergic LGICs in the nervous system, we generated reporter lines to characterize their neural expression patterns. We used a similar set of fluorescent reporter lines to characterize the expression pattern of the newly deorphanized LGICs using transcriptional reporter transgenes in which the upstream promoter of the *lgc* gene drove the expression of a fluorescent protein. We then identified transgene expression based on location, morphology, and coexpression with known marker lines. Using such transcriptional reporters, we observed primarily neuronal expression of the genes in the LGC-57 group, with little overlap observed in the neurons that were expressing reporters for *lgc-40*, *lgc-57*, and *lgc-58* (Fig. 4*A–C*). *lgc-40* was expressed in many pharyngeal neurons (M2, M3, MC, MI, I2), *lgc-57* in the A-class and B-class motorneurons of the ventral cord (VC), and *lgc-58* in the egg-laying motorneurons (*lgc-57* was also observed in a subset of VCs). This suggests that these channels are likely to exist primarily as homomers *in vivo* and function in distinct target neurons. Further, we observed the reporter for *lgc-39* in a range of interneurons and motor neurons, including the AVA premotor interneurons (Fig. 4*E*). In addition to receiving extensive cholinergic input, the AVA neurons are the major synaptic target for the only octopaminergic neurons, the RICs, suggesting that LGC-39 may be exposed to both octopamine and ACh *in vivo* (Fig. 4*F*) and may be involved in both cholinergic and octopaminergic synaptic transmission. Finally, we found that the ACC group channel, *lgc-49,* was expressed in sensory neurons, including posterior sensory neurons such as ALN and PLN (Fig. 4*D*).

10.1523/JNEUROSCI.1516-22.2022.f4-1 Figure 4-1 Expression patterns of still orphan LGICs and LGC-46 characterization. ***A***, ***B***, Expression of fluorescent reporters for still orphan LGCIs, head body, and tail are shown for *lgc-42* and *lgc-47* (***A***), head only is shown for *lgc-43*, *lgc-45*, and *lgc-48* (***B***). ***C***, Continues recording trace for LGC-46 and the ACh-induced dose–response curve for LGC-46. Error bars represent SEM of 6 oocytes. Insert shows EC_50_ values. ***D***, Expression of fluorescent reporters for lgc-46p shows a broad neuronal expression pattern with expression in, for example, AIZ, RIH, and AVE neurons. Download Figure 4-1, TIF file.

10.1523/JNEUROSCI.1516-22.2022.tab4-1 Table 4-1 List of *C. elegans* strains used in this study. Download Table 4-1, DOCX file.

10.1523/JNEUROSCI.1516-22.2022.tab4-2 Table 4-2 List of primers used in this study. Download Table 4-2, DOCX file.

10.1523/JNEUROSCI.1516-22.2022.tab4-3 Table 4-3 Expression overlap between fluorescent reporter strains and CeNGEN RNAseq expression data. Download Table 4-3, XLSX file.

We also used reporters to analyze the expression pattern of several still-orphan LGICs, from the diverse and ACC groups, including *lgc-42*, *lgc-47*, *lgc-48*, *lgc-43*, and *lgc-45*, as well as the previously deorphanized ACh-gated channel *lgc-46* (Takayanagi-Kiya et al., 2016; Extended Data Fig. 4-1*A–D*). These reporters also showed diverse and distinct patterns of expression, primarily in neurons. For example, *lgc-46* was broadly expressed in several neurons, mostly in the head (Extended Data Fig. 4-1*D*). Most of the orphan channels were also expressed specifically in neurons; for *lgc-47*, this expression was unusually broad, encompassing sensory neurons, motor neurons, and interneurons (Extended Data Fig. 4-1*A*). Interestingly, we noted that the expression of *lgc-47* overlaps with the reported single-cell RNAseq expression profile of *acc-1* in several classes of motor neurons (Taylor et al., 2021), such as the SMDs, RMDs, M3, and DA neurons (Extended Data Table 4-3). This, in combination with the functional data from coexpressing these in *Xenopus* oocytes (Extended Data Fig. 2-1*F*,*G*), may suggest that LGC-47 and ACC-1 are able to form a heteromeric channel. In contrast, *lgc-48* was expressed only in a single pair of neurons, the ADL chemosensory neurons (Extended Data Fig. 4-1*B*). Interestingly, the two orphan channels, *lgc-43* and *lgc-45*, both of which lack a PAR sequence and may thus encode cationic channels (Extended Data Fig. 1-3*A*), did not appear to be expressed in any neuronal tissue, but instead in the hypodermis (Extended Data Fig. 4-1*B*). In general, our reporter expression patterns aligned well with single-cell RNAseq data from the CeNGEN project (Taylor et al., 2021; Extended Data Table 4-3). Together, these data suggest that these channels play various roles inside, and outside, the nervous system.

### Excitatory and inhibitory ionotropic acetylcholine receptors are coexpressed in many neurons

Our fluorescent reporter expression analysis indicated that many of the inhibitory acetylcholine-gated channels newly deorphanized in this study are expressed in neurons previously shown to also express excitatory acetylcholine-gated channels (Raizen et al., 1995; Barbagallo et al., 2010). These results imply that acetylcholine may have a larger role as an inhibitory neurotransmitter than previously appreciated, and that acetylcholine contributes to both inhibitory and excitatory events in many neurons. To determine the extent to which excitatory and inhibitory ionotropic receptors for the same neurotransmitter are coexpressed in individual neural classes, we made use of the single-cell RNAseq dataset from *C. elegans* neurons (Taylor et al., 2021). We first generated a complete list of ionotropic receptors for each of the three classical neurotransmitters acetylcholine, GABA, and glutamate (Extended Data Table 5-1). Since channels with unknown ligand identity would have the potential to bias predictions, we predicted the ligand and ion selectivity of orphan channels based on homology with closely related characterized channels, and the presence, or absence, of a PAR motif in the M1-2 intracellular loop (see Materials and Methods).

From this analysis, we found a remarkable frequency of neural classes that coexpress both inhibitory and excitatory ionotropic receptors for the same neurotransmitter. This was particularly notable for acetylcholine, for which >60% of the neural classes expressed both excitatory and inhibitory acetylcholine-gated channels. In contrast, GABA-gated channels were more biased toward inhibition, with only 9% of neural classes expressing both types of receptors and >40% of neural classes expressing only inhibitory GABA-gated channels (Fig. 5*A*, Extended Data Fig. 5-1). To make generalized predictions of synaptic polarity, we summed expression of inhibitory and excitatory ionotropic receptors for each neurotransmitter, in each neural class and assigned synapse polarity based on the most prevalent receptors in each neural class, assuming that all receptors in a cell are present equally at all synapses (Fig. 5*C*, Extended Data Fig. 5-2). This approach does not take heteromerization of different subunits or differences in synaptic strength into account and should therefore be considered a generalized prediction. The analysis suggested that the majority of acetylcholine and glutamate synapses are excitatory, and most GABAergic synapses are inhibitory, though this varied significantly for individual connections (Fig. 5*B*).

10.1523/JNEUROSCI.1516-22.2022.f5-1 Figure 5-1 Binary heatmap of synaptic sign prediction for the three major neurotransmitters, ACh, glutamate, and GABA. The heatmap shows the summed expression level of all LGICs in *C. elegans* per neural class and neurotransmitter, a net sum of excitatory channels is displayed in red, inhibitory in green, equal expression in peach, and no expression of LGICs in white. Download Figure 5-1, TIF file.

10.1523/JNEUROSCI.1516-22.2022.f5-2 Figure 5-2 Expression heatmap for the three major neurotransmitters. The heatmap shows the summed expression level and ion selectivity for LGICs separated by transmitter [ACh, glutamate (Glu), and GABA] and neural class. Net excitatory channel expression is represented in pink, and inhibitory in green. Download Figure 5-2, TIF file.

10.1523/JNEUROSCI.1516-22.2022.tab5-1 Table 5-1 The list shows ligand identity and ion selectivity for LGICs in *C. elegans*. Each gene is assigned a polarity based upon its ion selectivity; this is shown in column Pos/Neg as follows: P, positive (cation selectivity); or N, negative (anion selectivity). For orphan receptors, the ligand and ion selectivity have been predicted based on homology; this has been noted in the column Inferred as yes. The references where the receptor has a validated ligand are from experiments evaluating receptors using electrophysiological characterization in a heterologous expression system. Download Table 5-1, DOCX file.

To examine the validity of our polarity predictions, we investigated the sign prediction using previously characterized neuronal circuits. We picked the well studied locomotion circuit (Chalfie et al., 1985) consisting of the interneurons AVD, AVE, and AVA, which initiate reversals, and PVC and AVB that initiate forward movement. Most of our predicted connection polarities (Fig. 5*D*) were consistent with circuit data from previous studies, such as the excitatory connection between AVA and the VA and DA motor neurons, which is involved in controlling reverse locomotion, as well as the excitatory connection from the sensory neuron ASH to the reverse command neuron AVA (Mellem et al., 2002; Piggott et al., 2011). We also observed predicted connections that appeared counterintuitive such as an inhibitory acetylcholine connection from AVD to AVA, two interneuron pairs thought to be coordinately active during reverse locomotion (Faumont et al., 2012; Fig. 5*D*). While some studies have proposed additional inhibitory connections within this circuit (Rakowski and Karbowski, 2017), AVA neurons express several acetylcholine-gated channels and have a relatively low ratio of inhibitory to excitatory ionotropic receptor expression (1:3), on which this prediction was made. This suggests that some connections may indeed be both inhibitory and excitatory, especially where a neuron expresses a large range of different channels and receives input from many different neural classes. Connections such as these require further *in vivo* investigations to address these predictions.

### Determining synaptic localization of LGICs

We reasoned that the single-cell RNAseq dataset (Taylor et al., 2021) might also be useful for predicting the intracellular localization of cholinergic LGICs, as presynaptic ionotropic receptors would be predicted to be expressed in cholinergic neurons, while postsynaptic ionotropic receptors should be expressed in neurons receiving cholinergic input. To assess the correlation between the number of acetylcholine synapses, a neuron makes (“outgoing ACh synapses”) or receives (“incoming ACh synapses”), with the expression level of cholinergic LGICs, we produced two heatmaps showing the expression of acetylcholine-gated channels, with neural classes ranked by the total number of incoming or outgoing acetylcholine synapses (Fig. 6*A*,*B*). This analysis highlights that the expression of some ACC group channels, in particular *lgc-46*, correlates with the number of both incoming and outgoing ACh synapses (Fig. 6*C*,*D*, Extended Data Fig. 6-1), which is in line with previous studies describing both a presynaptic and postsynaptic role for LGC-46 (Takayanagi-Kiya et al., 2016; Liu et al., 2017). This correlation suggests that these channels may be acting either presynaptically or postsynaptically. In contrast, members of the choline-gated and acetylcholine-gated LGC-57 group, *lgc-40*, *lgc-57*, and *lgc-58*, showed little correlation with either incoming or outgoing synapses (Fig. 6*C*,*D*). Surprisingly for this subgroup, several cells with high acetylcholine connectivity showed low channel expression level (Fig. 6*C*,*D*). This may be suggestive of an extrasynaptic role for these channels; however, further evidence is required to assess these assumptions.

10.1523/JNEUROSCI.1516-22.2022.f6-1 Figure 6-1 Correlation graphs between cholinergic synapses and expression of selected LGICs. Scatter plots showing gene expression level versus the total number of incoming cholinergic synapses (red) and outgoing cholinergic synapses (orange). Lines fit using relplot with shaded areas representing the SE of the line fit. Download Figure 6-1, TIF file.

**Figure 7.**
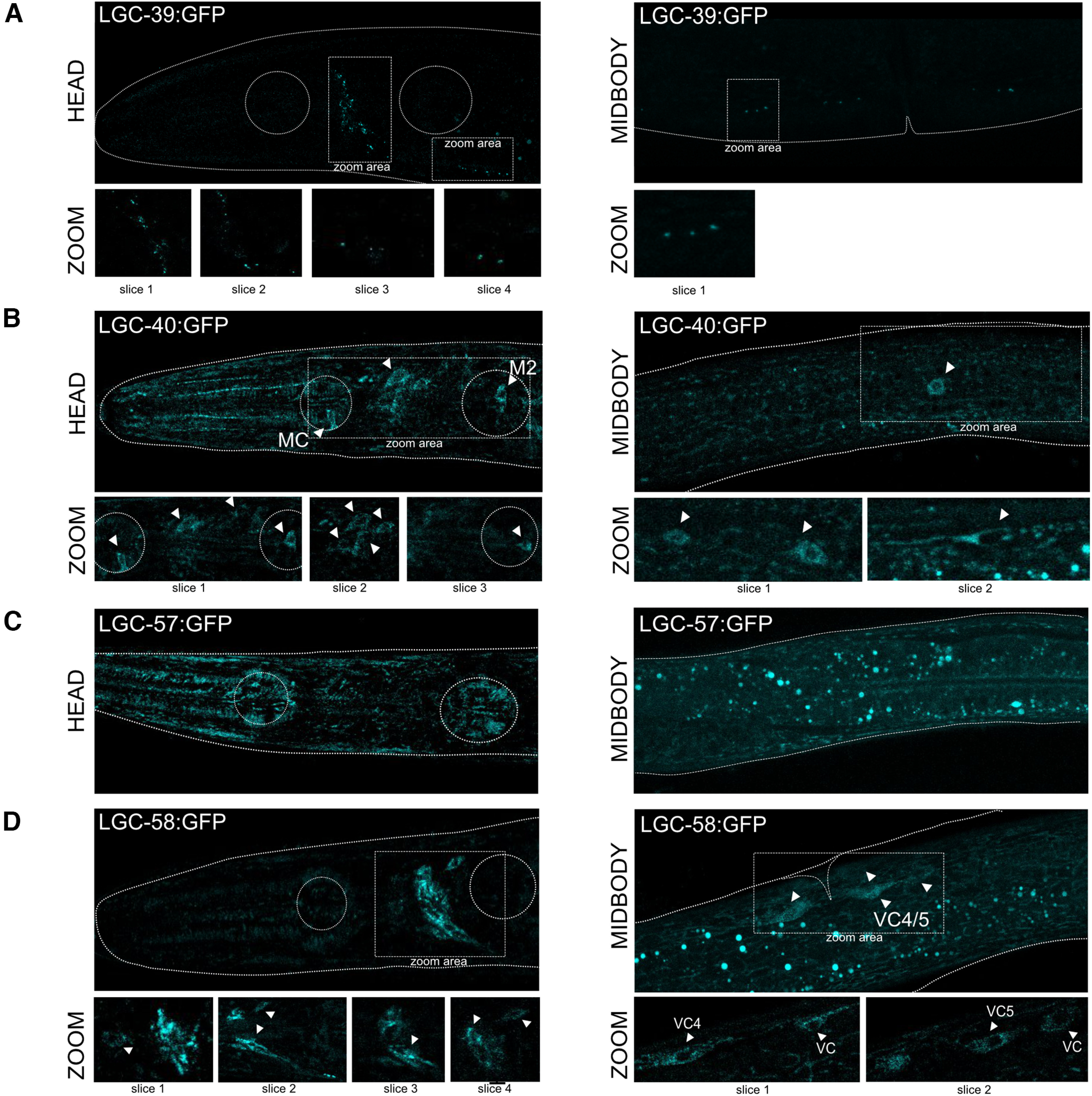
Protein expression pattern of cholinergic ion channels. ***A–D***, Localization of endogenously GFP tagged LGC-39, LGC-40, LGC-57, and LGC-58. White arrows highlight areas of interest, represented in higher magnification below (Extended Data Fig. 7-1, dose–response traces for GFP tagged channels).

To empirically assess the synaptic localization of cholinergic LGICs, we generated endogenous GFP-tagged CRISPR lines for members of the LGC-57 subgroup, including *lgc-39*, *lgc-40*, *lgc-57*, and *lgc-58* (Fig. 7*A–D*). In all cases, GFP was inserted in the intracellular M3/4 loop, and the function of the resulting chimeric protein was verified in *Xenopus* oocytes (Extended Data Fig. 7-1*A*). We observed a clear difference in the localization pattern for these channels. LGC-39::GFP was localized in distinct punctate structures both in the nerve ring and along the ventral cord, suggestive of synaptic localization (Fig. 7*A*) and consistent with the positive correlation between *lgc-39* expression and incoming and outgoing acetylcholine synapses (Fig. 6*A–D*). Members of the choline-gated LGC-57 group, however, showed diffuse protein expression. LGC-40::GFP appeared to have diffuse expression in the nerve ring, and touch receptor neurons, with cell bodies often being visible (Fig. 7*B*). Notably, cell body LGC-40::GFP expression was detected in the posterior and anterior bulbs in cells that anatomically correspond to MC and M2 neurons (Fig. 7*B*), while LGC-57::GFP appeared to have overall low expression and little protein localization could be seen above background (Fig. 7*C*). LGC-58::GFP was clearly visible in the nerve ring and VC4/5, including some punctate structures (Fig. 7*D*). Since these choline-sensitive members of the LGC-57 group showed little correlation with acetylcholine synapses, their diffuse protein localization may be indicative of an extrasynaptic role (Figs. 6*C*, 7*D*).

10.1523/JNEUROSCI.1516-22.2022.f7-1 Figure 7-1 GFP tagging of LGICs does not influence channel function. ACh- or choline-induced dose–response curves for oocytes expressing GFP tagged versions of LGC-40, LGC-57, and LGC-58 shows that EC_50_ values are not influenced by the insertion of the GFP tag. Error bars represent SEM of 7–12 oocytes per construct, insert shows EC_50_ values. Download Figure 7-1, TIF file.

## Discussion

### A novel family of cholinergic LGICs

This study highlights the diversity among cholinergic LGICs in *C. elegans*. Nematodes have previously been shown to express acetylcholine-gated and choline-gated excitatory LGICs related to nicotinic receptors, as well as inhibitory acetylcholine-gated chloride in the ACC group. Here we describe a second inhibitory subfamily that contains channels gated by both choline and ACh: LGC-40, LGC-57, and LGC-58 (previously named GGR-1 and GGR-2). In contrast to the ACC group of acetylcholine-gated anion channels, these newly deorphanized channels are gated preferentially by choline, the metabolite of acetylcholine which is abundant at cholinergic synapses.

These results add to the already extensive catalog of acetylcholine-gated channels in *C. elegans* (Putrenko et al., 2005; Takayanagi-Kiya et al., 2016) and to the growing number of choline-gated channels described in *C. elegans,* which previously consisted of the excitatory DEG-3/DES-2 channel found within the nAChR superfamily (Yassin et al., 2001). Together with our new data, this highlights the expansion and importance of cholinergic transmission in nematodes. These newly deorphanized channels display subtle variations in their ability to bind ligands and antagonists, which translates into physiologically relevant differences that may increase the fine tuning in the control of neuronal transmission and contribute to complex neuronal signaling within a relatively minimal neuronal network. Interestingly, the electrophysiologically similar channels LGC-40, LGC-57, and LGC-58 show largely distinct patterns of expression within the nervous system of *C. elegans*, suggesting that they may form homomeric channels with distinct functions *in vivo*. When tagged with a fluorescent protein, these three channels also showed a diffuse localization pattern within the neuron, suggesting that in contrast with the ACC group channels, such as LGC-46 (Takayanagi-Kiya et al., 2016; Liu et al., 2017), these channels may not be synaptically localized. This suggests a possible distinct extrasynaptic role for choline acting via these channels in the modulation of neural activity.

The observation that choline, a molecule generated at cholinergic synapses through catabolism of acetylcholine by cholinesterases, shows higher efficacy for LGC-40, LGC-57 and LGC-58 raises the possibility that choline is their true *in vivo* ligand and that choline itself may function as a neuromodulator. The idea that choline could activate cholinergic receptors differently from acetylcholine has been discussed for other cholinergic receptors that can be dose-dependently blocked or activated by choline (Purohit and Grosman, 2006). Here we have identified ionotropic cholinergic receptors in which choline acts as a full agonist, showing preference in activation by choline over acetylcholine. Previous reports suggest that aromatic residues in the extracellular domain of mammalian neuromuscular AChRs play a vital part in stabilizing the binding of acetylcholine over the binding to choline (Bruhova and Auerbach, 2017). Interestingly, although aromatic residues are present in the putative ligand binding regions of the *C. elegans* choline-gated channels identified in this study, their positions vary in comparison to mammalian AChRs (Extended Data Fig. 1-3). Specifically, the LYS165–TYR210 hydrogen bond thought to be important in specifying acetylcholine over choline binding in mouse α 1 (*Chrna1*; Bruhova and Auerbach, 2017) appears to be replaced with a hydrogen bond between ARG183 and TYR229 in LGC-57, which is conserved in both LGC-40 and LGC-58; in addition, a further key tyrosine residue in mouse α 1 (TYR218) is replaced by tryptophan in all three choline-gated channels (Extended Data Figs. 1-3, 1-4). We also note that none of the acetylcholine-activated or choline-activated channels characterized in this study contain the vicinal cysteine residues (Extended Data Figs. 1-3, 1-4) that are characteristic of nAChRs (Kao and Karlin, 1986). The regulation of choline concentrations in the context of acting as a neuromodulator in *C. elegans* is unclear. In mammals studies have shown that reuptake of choline at the synapse may occur less often than previously thought (Muramatsu et al., 2017), and the regulation of choline reuptake is highly plastic (Ferguson et al., 2004). Taken together, it is not unreasonable to hypothesize that choline could be an authentic endogenous ligand for these channels *in vivo*.

### A polymodal LGIC activated by both aminergic and cholinergic ligands

In this study, we also identified a novel polymodal channel, LGC-39, which was gated not only by acetylcholine, but also by the aminergic ligands tyramine and octopamine. We observed dose-dependent activation of LGC-39 channels by these structurally dissimilar ligands at similar, physiologically relevant concentrations. Interestingly, while both cholinergic and noncholinergic antagonists blocked the acetylcholine-induced response of LGC-39, only yohimbine, an α2 adrenergic receptor inhibitor, was able to block the octopamine-induced response. Despite being an inhibitor of octopaminergic GPCRs in *C. elegans* (Packham et al., 2010), epinastine was not able to inhibit the octopamine-induced response, instead it acted as an agonist both alone and in the presence of octopamine. It has previously been shown that strychnine acts as an agonist on mutant forms of α7 nAChRs in which residues contributing to acetylcholine binding were altered (Palma et al., 1999). It is possible that strychnine binds in a similar manner to wild-type LGC-39, thereby disrupting acetylcholine activation but not octopamine activation, suggesting that acetylcholine and octopamine have different binding modes.

The expression pattern of *lgc-39* suggests that the channel might be exposed to all these ligands *in vivo*. For example, *lgc-39* is highly expressed in the AVA premotor neurons, which receive a large amount of input from acetylcholine producing neurons (White, 1986), as well as from the RIC neurons, the only octopamine-producing cells in the *C. elegans* nervous system (Alkema et al., 2005). The AVA neurons also receive some input from tyraminergic and dopaminergic neurons, transmitters that we also found can activate LGC-39. Interestingly, in contrast to the choline-gated channels LGC-40, LGC-57, and LGC-58, we observe clear punctate localization of LGC-39 in both the nerve ring and along the ventral cord, with no fluorescence visible in the cell bodies. This may suggest a role for LGC-39 as a postsynaptic receptor for both cholinergic and aminergic neurotransmission.

The concept of a truly polymodal ionotropic receptor that can be activated by structurally diverse compounds has not been investigated before in great detail, though previous observations have described roles for receptors that can use dual ligands for allosteric modulation (Cummings and Popescu, 2015). For example, dopamine exhibits a pseudocompetitive ability to antagonize GABA_A_ currents, although this effect cannot be blocked by competitive GABA_A_ antagonists, which bind the main binding pocket (Hoerbelt et al., 2015). Further, d-serine has been shown to function as an allosteric modulator of NMDA receptor activity (Wolosker and Balu, 2020). In contrast to these examples, based on their capability to achieve dose-dependent activation by both amines and acetylcholine, both groups of neurotransmitters appear to be true ligands of LGC-39, most likely interacting with the ligand binding domain. Understanding the mechanisms by which these multiple neurotransmitters can activate LGC-39 and potentially affect different behavioral outputs will be of interest in future studies.

### Functional insights into the *C. elegans* connectome

With increasing molecular and physiological characterization of neurotransmitter receptors in *C. elegans*, it is becoming feasible to predict the functionality of synapses more accurately in the *C. elegans* connectome. In this study, we used the expression patterns of newly and previously deorphanized LGICs for the three classical neurotransmitters, acetylcholine, glutamate, and GABA, to predict the polarity of synapses in the *C. elegans* connectome. By assigning synapse polarity based on relative expression levels of anionic and cationic receptors, we have provisionally predicted the sign of chemical synapses involving classical neurotransmitters. Although similar attempts to assign polarity to *C. elegans* synapses have been made in the past (Fenyves et al., 2020), these predictions were based on incomplete or incorrect ligand assignment for many LGICs, for example *ggr-1 (lgc-57)*, *ggr-2 (lgc-58)*, and *ggr-3 (lgc-56)*, were listed as anionic GABA receptors, whereas we subsequently have shown that *lgc-57* and *lgc-58* are gated by choline and ACh and *lgc-56* by monoamines (Morud et al., 2021). Our revised predictions correlate well with experimental data for many well characterized circuits, such as the excitatory connections between the ASH nociceptors and the AVA interneurons, as well as between the AVAs and the VA and DA motorneurons (Mellem et al., 2002; Piggott et al., 2011). Our predicted inhibitory connection between AVB and AVA interneurons also corresponds well with empirical data on the locomotor circuit (Kawano et al., 2011; Qi et al., 2012). In addition to providing sign predictions for synaptic connections, our model also provides a ratio of excitatory to inhibitory expression for each neuronal class and neurotransmitter. Not only do our predictions generate interesting functional hypotheses for future investigation, but this additional information also allows these predictions to be critically assessed. It also raises the question of whether these connections, with low receptor ratios, represent truly complex connections, a question that could be addressed in future studies.

A surprising outcome of the expression analysis was the high frequency with which individual neurons expressed cationic and anionic receptors for the same neurotransmitter. This was especially prevalent for acetylcholine; our analysis indicated that 60% of neural classes express both inhibitory and excitatory ionotropic receptors for acetylcholine, 30% for glutamate, and 10% for GABA. One explanation for this apparent paradox is that excitatory and inhibitory ionotropic receptors might be differentially localized in neurons, with some found extrasynaptically and others enriched in synapses. Various *C. elegans* LGICs are known to act in regions other than the postsynapse; for example, *lgc-35* has been shown to mediate GABA spillover transmission (Jobson et al., 2015), while *lgc-46* appears be localized to both presynapses (Takayanagi-Kiya et al., 2016) and postsynapses (Liu et al., 2017). The choline-sensitive channels from the LGC-57 group likewise appear to be extrasynaptic in their localization (Fig. 7). In addition, postsynaptic sites might themselves contain a mixture of excitatory and inhibitory ionotropic receptors, which could differ in their ligand affinity, desensitization kinetics and regulation. Indeed, LGIC localization is not static; for example, glutamatergic AMPA receptors have been shown in many species to increase their synaptic localization during learning (Malinow and Malenka, 2002), and recent evidence indicates that *C. elegans* LGICs also display regulated membrane trafficking on learning (Morud et al., 2021). Thus, *C. elegans* may contain large numbers of complex cholinergic synapses with the potential to be excitatory or inhibitory depending on context or experience.
