# Supplementary figures and images for: A Novel and Functionally Diverse Class of Acetylcholine-Gated Ion Channels
Source: J Neurosci. 2023 Feb 15;43(7):1111–24. doi: 10.1523/JNEUROSCI.1516-22.2022 (PMC9962794; doi:10.1523/JNEUROSCI.1516-22.2022)

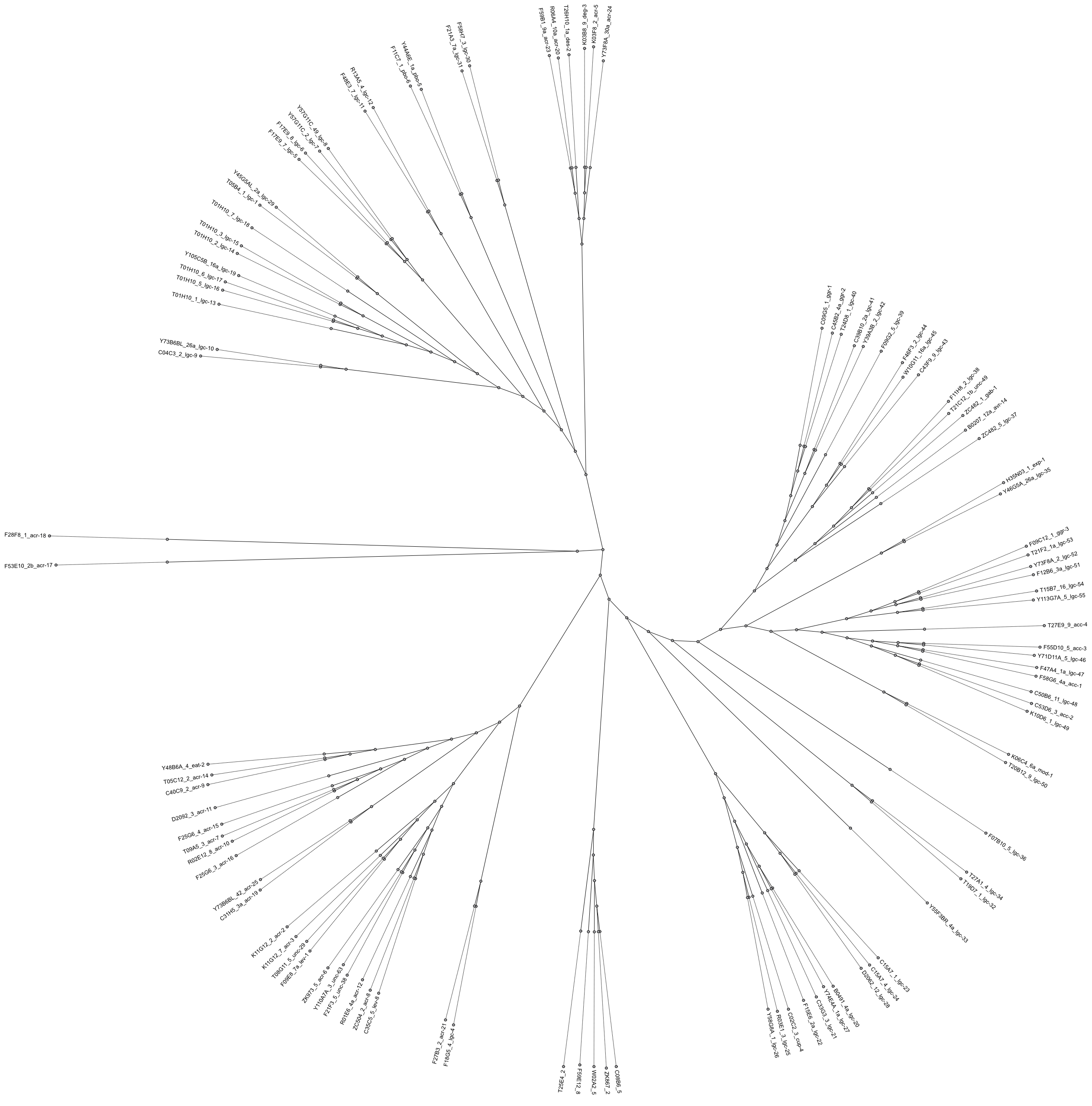

Supplement: Figure 1-1 — Full phylogenetic tree. Reproduced from the study by Morud et al. (2021). Generated with PHYLIP Neighbor Joining, not to scale. Download Figure 1-1, TIF file. [file ns-JN-RM-1516-22-s06.tif]

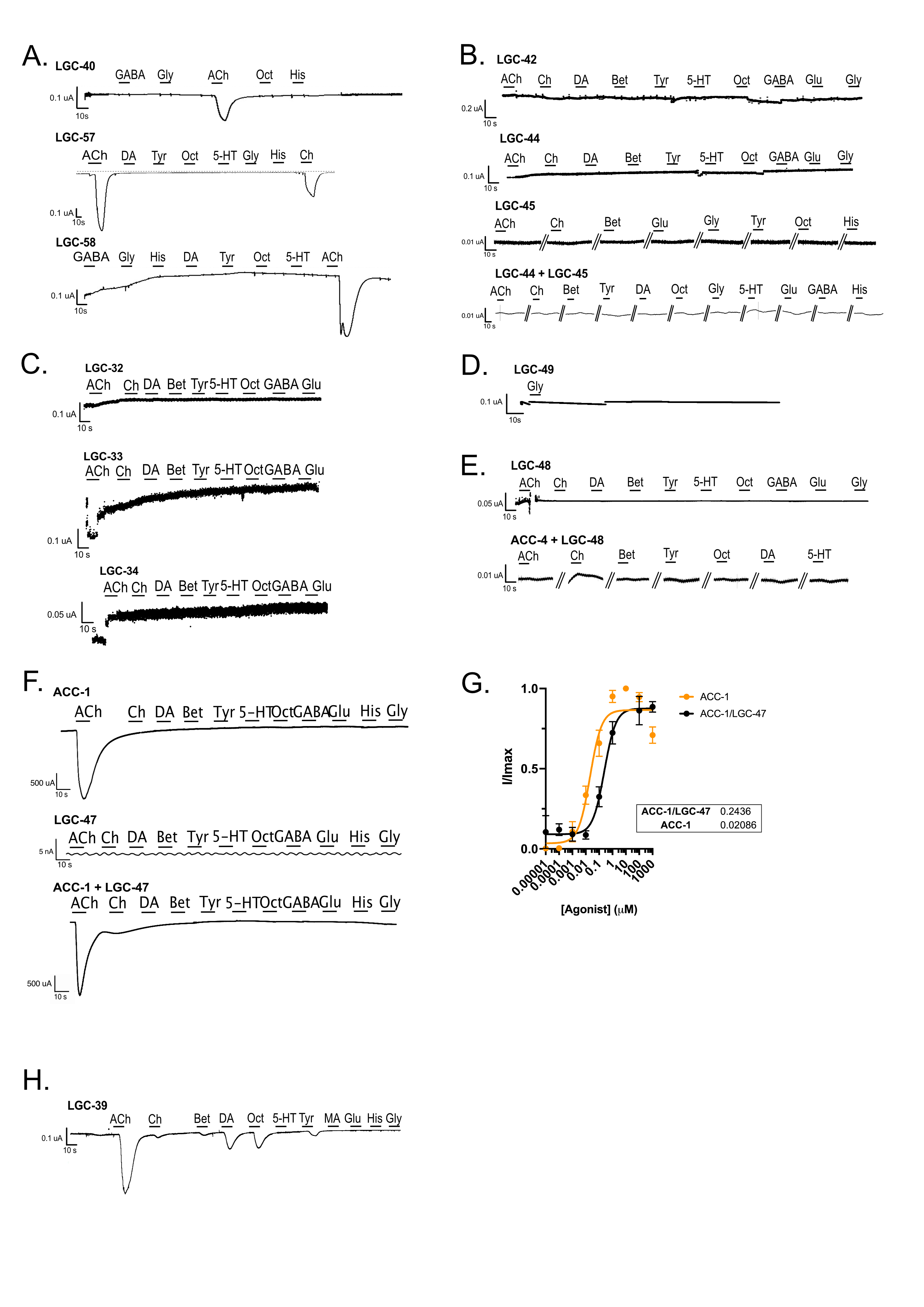

Supplement: Figure 1-2 — Negative traces for still orphan and characterized LGICs. A–F, H, Continuous TEVC traces from oocytes clamped at –60 mV expressing LGICs, exposed to 10 s of a selection of a panel of ligands. Ch, Choline; Bet, betaine; Tyr, tyramine; 5-HT, serotonin; Oct, octopamine; Gly, glycine; His, histamine; MA, melatonin. E, Coexpression of LGC-48 and ACC-4, and not LGC-48 on its own, did not show any agonist-induced current by the ligands tested. F, Continuous TEVC traces from oocytes clamped at –60 mV expressing LGC-47, ACC-1, or a combination, exposed to 10 s of a panel of ligands. Note that the small changes in current seen in the traces are attributed to recording and perfusion artifacts G, ACh-induced dose–response curves for oocytes expressing ACC-1 alone or in combination with LGC-47. Error bars represent the SEM of 7–12 oocytes/construct; insert shows EC50 values. H, Continuous TEVC traces from oocytes clamped at –60 mV expressing LGC-39 exposed to 10 s of a panel of ligands. Note that the small changes in current seen in the traces are attributed to recording and perfusion artifacts. Download Figure 1-2, TIF file. [file ns-JN-RM-1516-22-s07.tif]

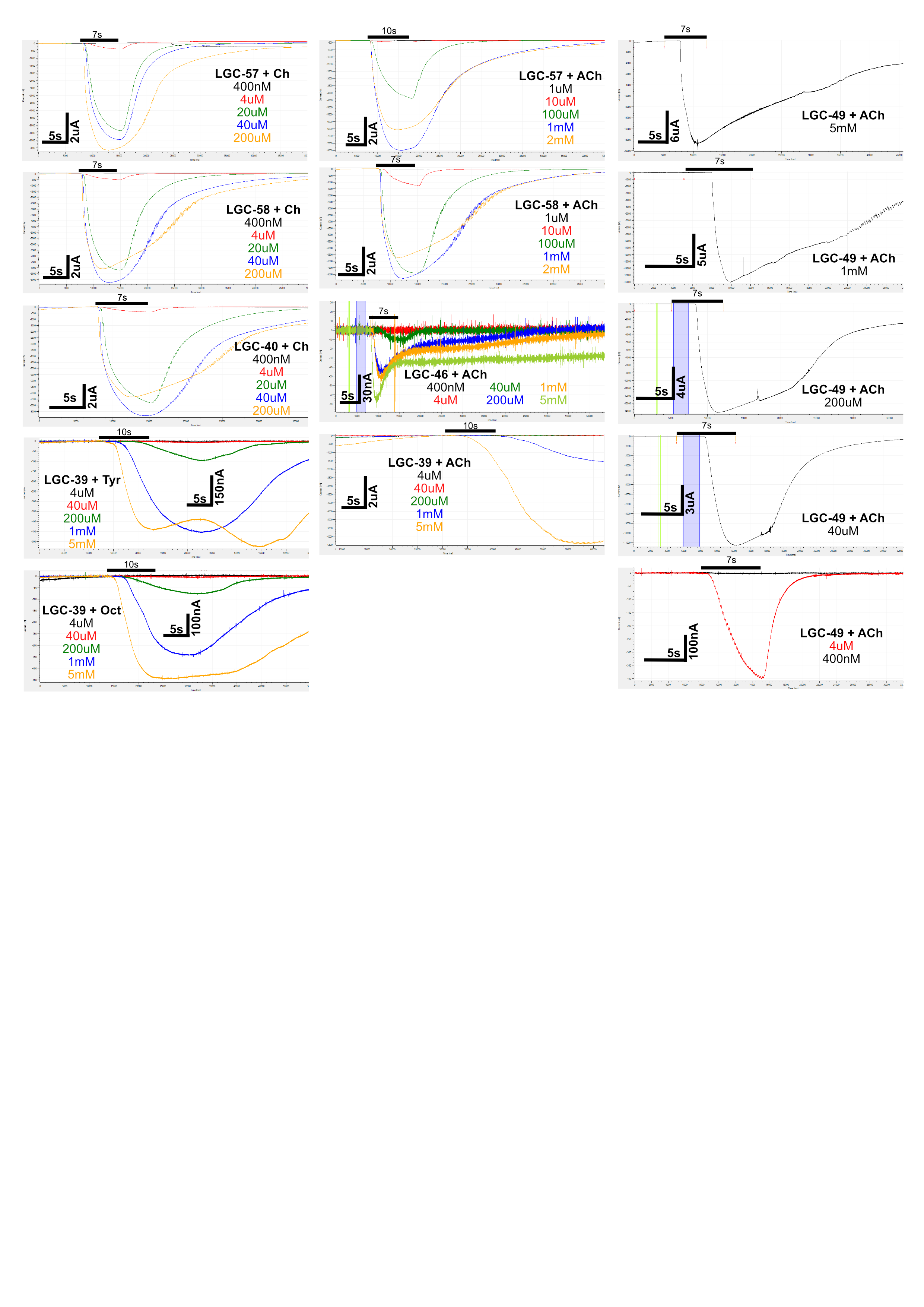

Supplement: Figure 1-5 — Representative traces of different doses during dose–response experiments for all characterized channels in this study. Black bars show agonist application time of either 7 or 10 s. Oct, Octopmaine; Tyr, tyramine. Download Figure 1-5, TIF file. [file ns-JN-RM-1516-22-s10.tif]

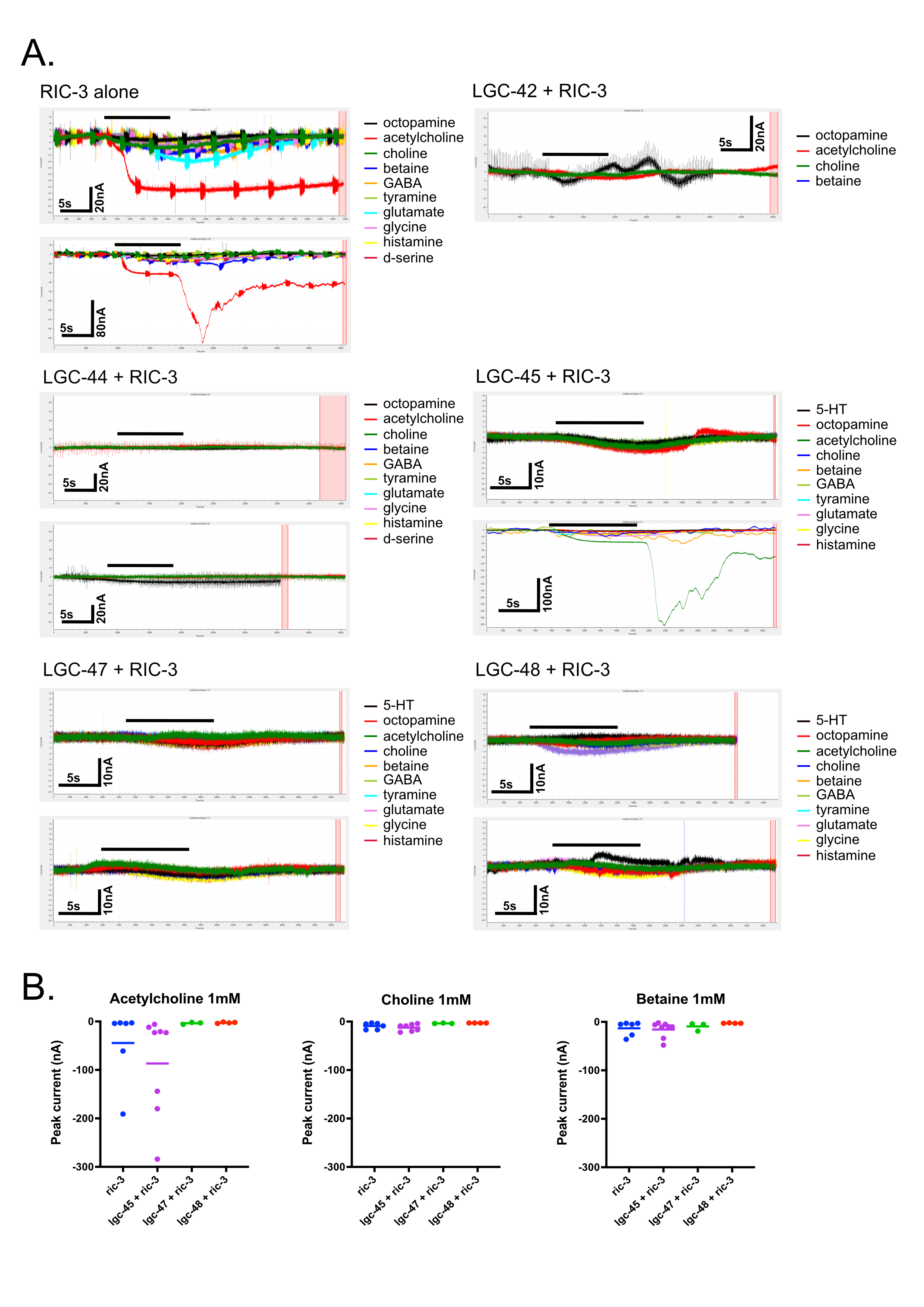

Supplement: Figure 1-6 — Still orphan LGICs coexpressed with RIC-3. A, Representative traces oocytes expressing RIC-3 alone or in combination with LGC-42, LGC-44, LGC-45, LGC-47, and LGC-48. Oocytes were exposed to a 10 s perfusion (indicated by the black bar) of selection of a panel of ligands. B, Quantification of peak current induced after perfusing at 1 mm acetylcholine, choline, and betaine. Download Figure 1-6, TIF file. [file ns-JN-RM-1516-22-s11.tif]

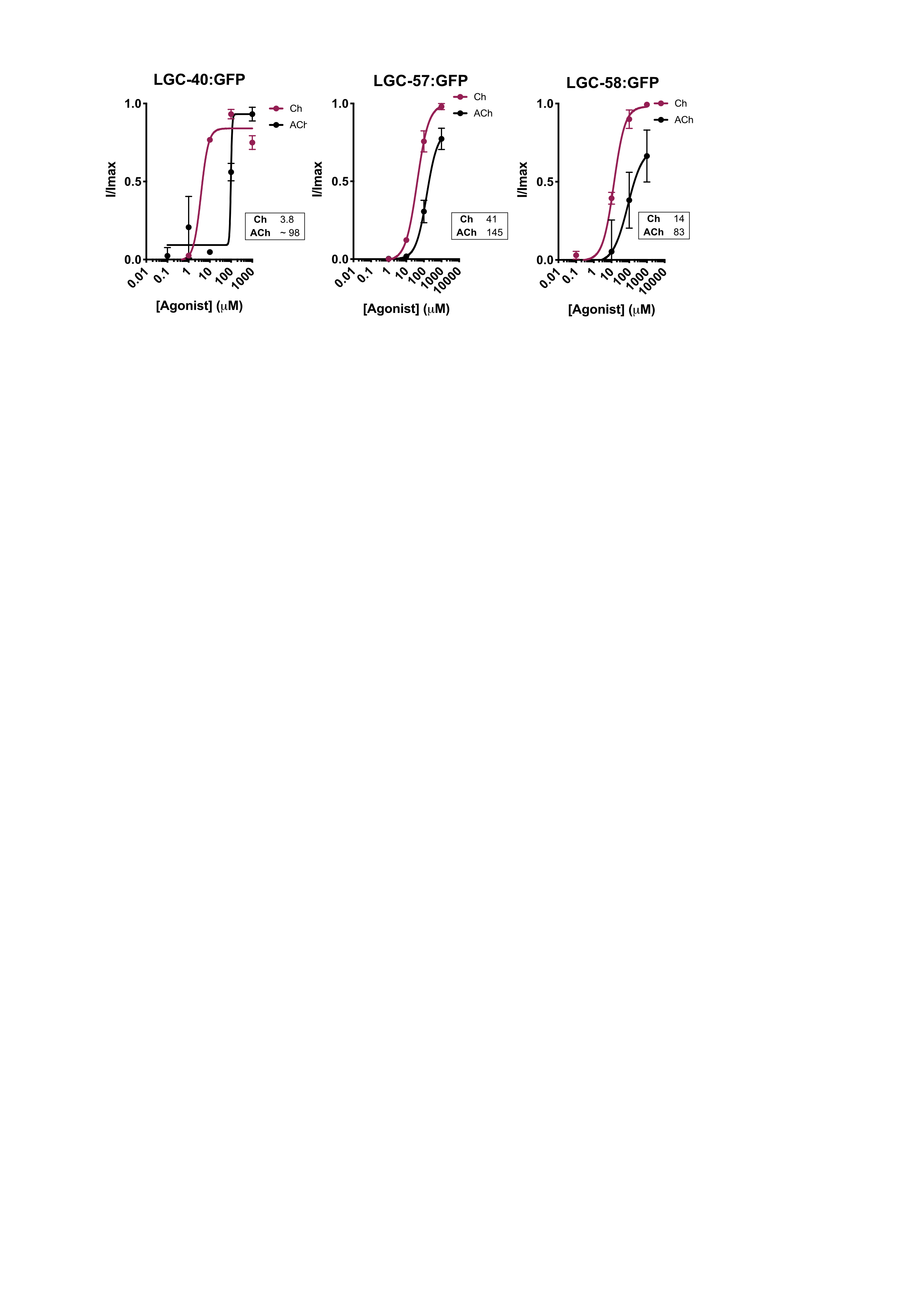

Supplement: Table 1-1 — Overview of LGICs and LGIC combinations screened in this study, including agonists/antagonists and selectivity information. Groups are highlight by color, as follows: ACC group of ACh-gated channels, red; LGC-45 group, yellow; LGC-41 group, blue; LGC-57 group, green. Download Table 1-1, TIF file. [file ns-JN-RM-1516-22-s17.tif]

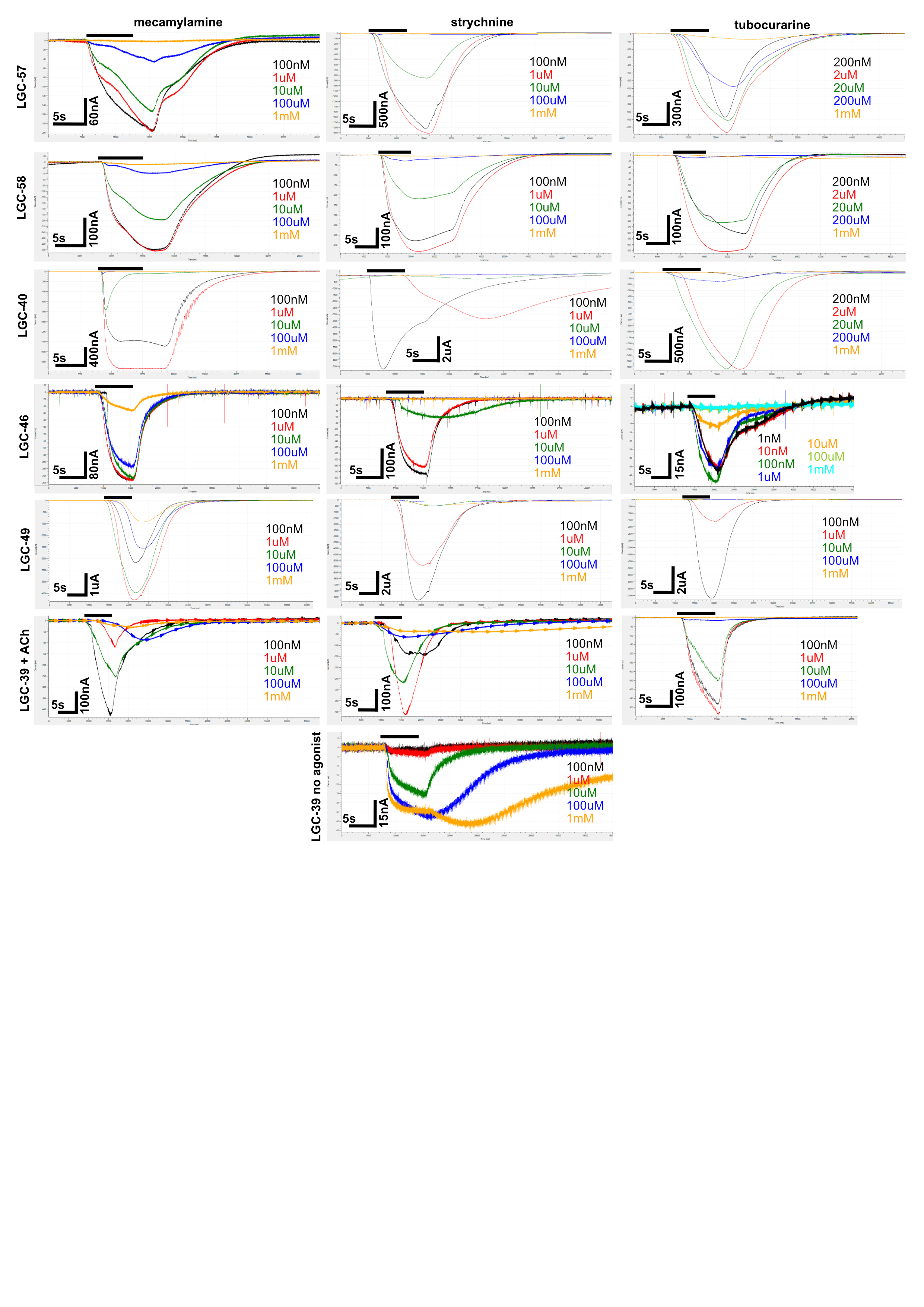

Supplement: Figure 2-1 — Representative traces during different antagonist applications. Each channel was exposed to its primary ligand at EC50 along with an increasing antagonist concentration. Black bars above the trace show an agonist/antagonist application time of 7 s. Download Figure 2-1, TIF file. [file ns-JN-RM-1516-22-s12.tif]

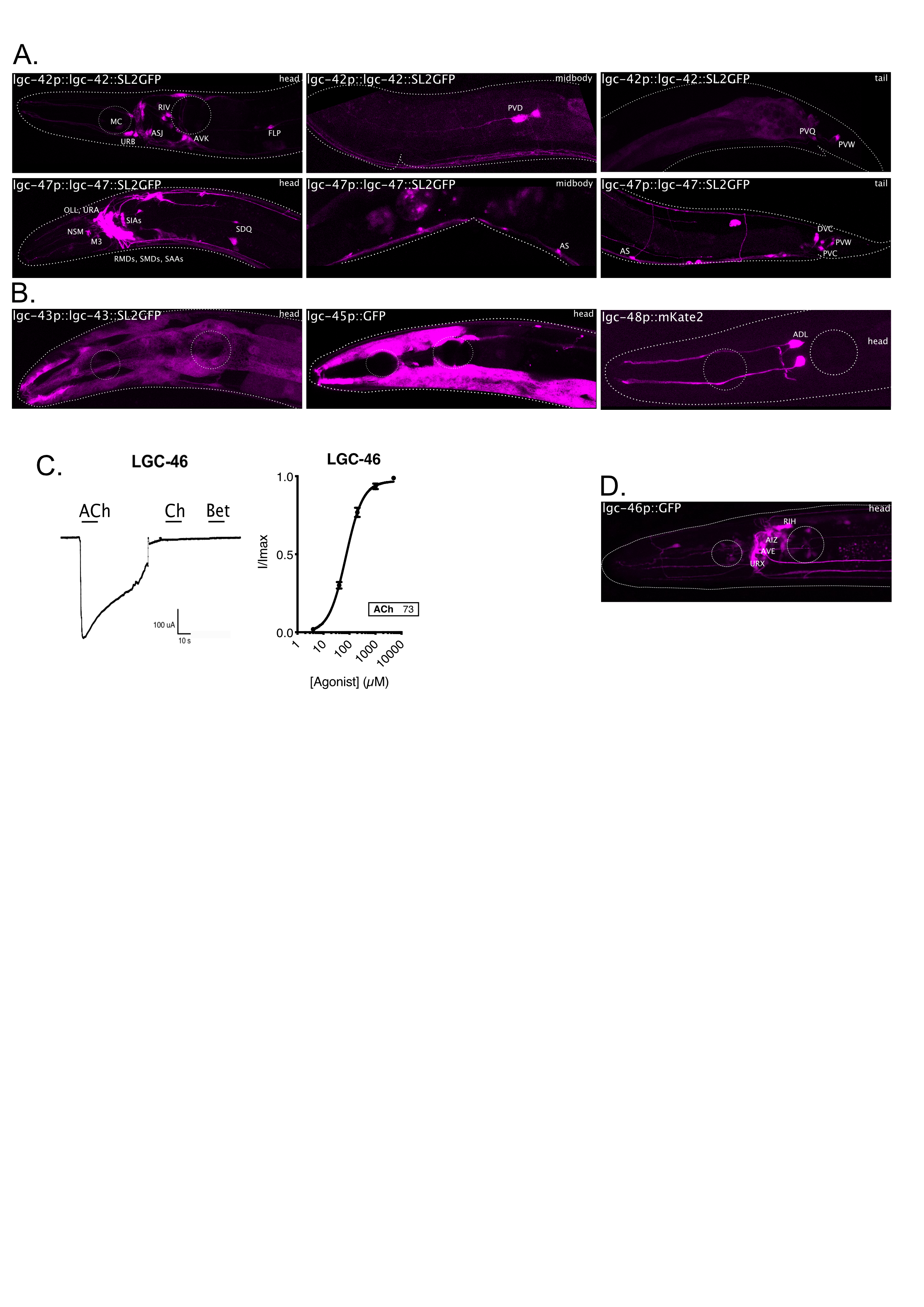

Supplement: Figure 4-1 — Expression patterns of still orphan LGICs and LGC-46 characterization. A, B, Expression of fluorescent reporters for still orphan LGCIs, head body, and tail are shown for lgc-42 and lgc-47 (A), head only is shown for lgc-43, lgc-45, and lgc-48 (B). C, Continues recording trace for LGC-46 and the ACh-induced dose–response curve for LGC-46. Error bars represent SEM of 6 oocytes. Insert shows EC50 values. D, Expression of fluorescent reporters for lgc-46p shows a broad neuronal expression pattern with expression in, for example, AIZ, RIH, and AVE neurons. Download Figure 4-1, TIF file. [file ns-JN-RM-1516-22-s13.tif]

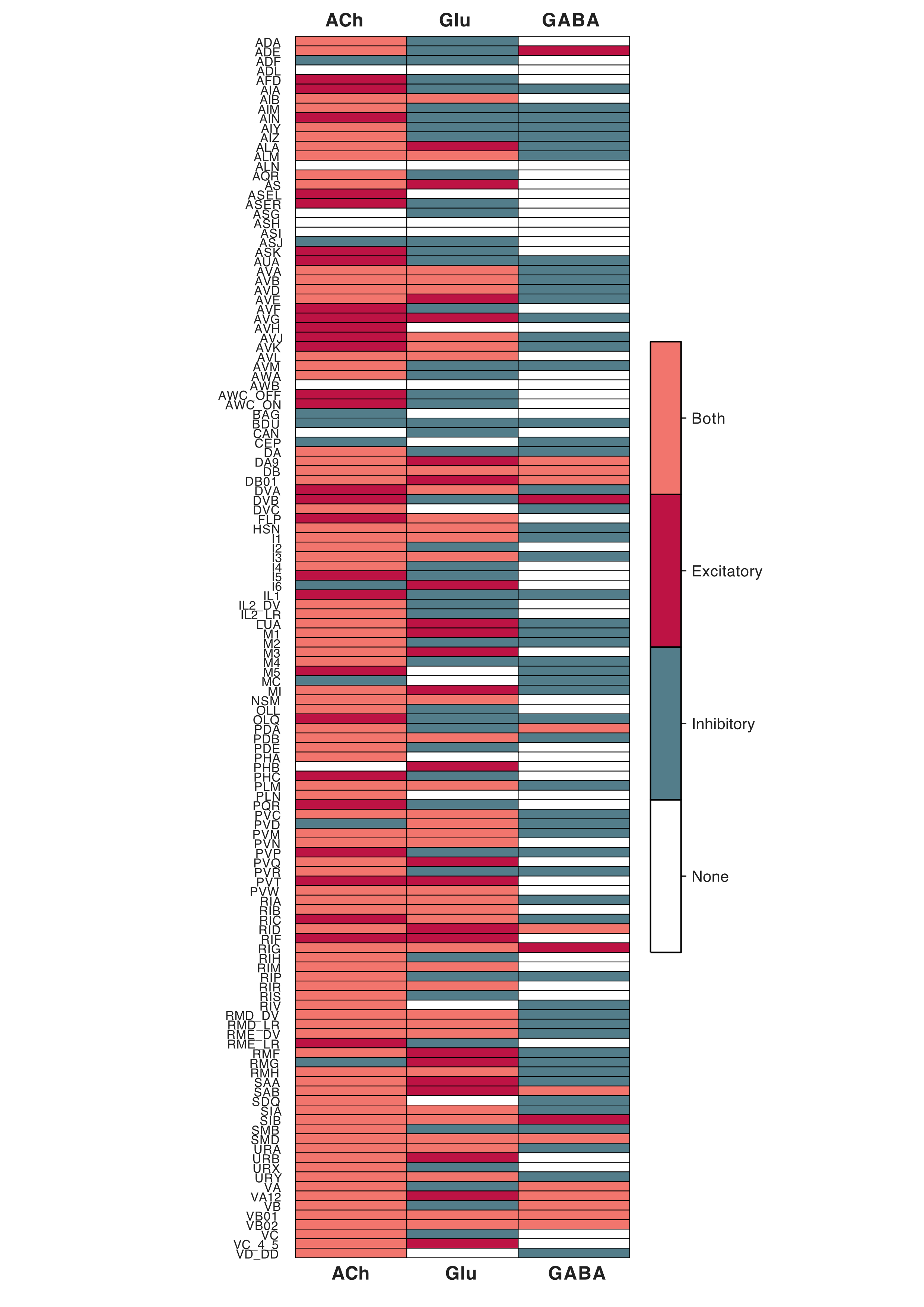

Supplement: Figure 5-1 — Binary heatmap of synaptic sign prediction for the three major neurotransmitters, ACh, glutamate, and GABA. The heatmap shows the summed expression level of all LGICs in C. elegans per neural class and neurotransmitter, a net sum of excitatory channels is displayed in red, inhibitory in green, equal expression in peach, and no expression of LGICs in white. Download Figure 5-1, TIF file. [file ns-JN-RM-1516-22-s14.tif]

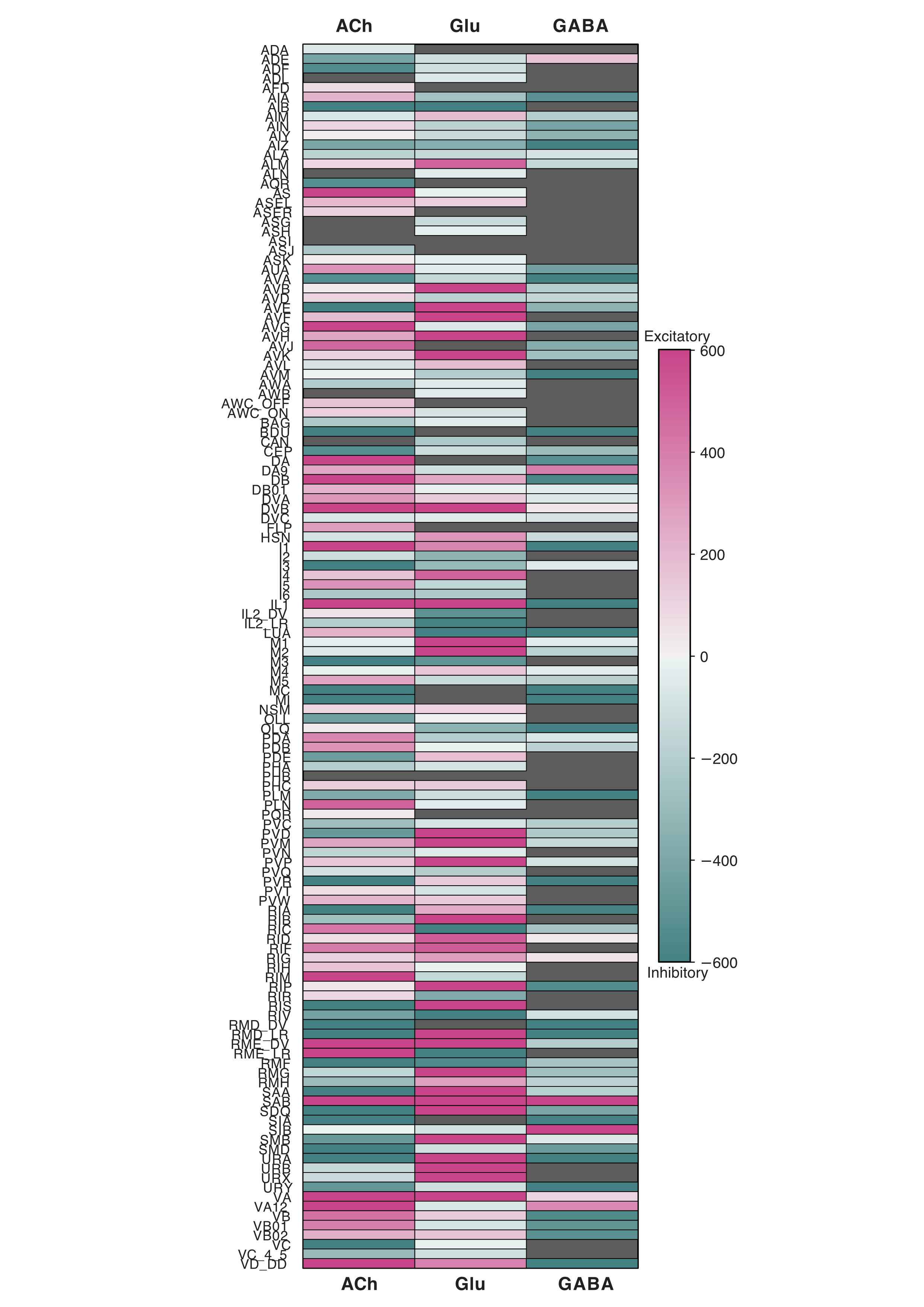

Supplement: Figure 5-2 — Expression heatmap for the three major neurotransmitters. The heatmap shows the summed expression level and ion selectivity for LGICs separated by transmitter [ACh, glutamate (Glu), and GABA] and neural class. Net excitatory channel expression is represented in pink, and inhibitory in green. Download Figure 5-2, TIF file. [file ns-JN-RM-1516-22-s15.tif]

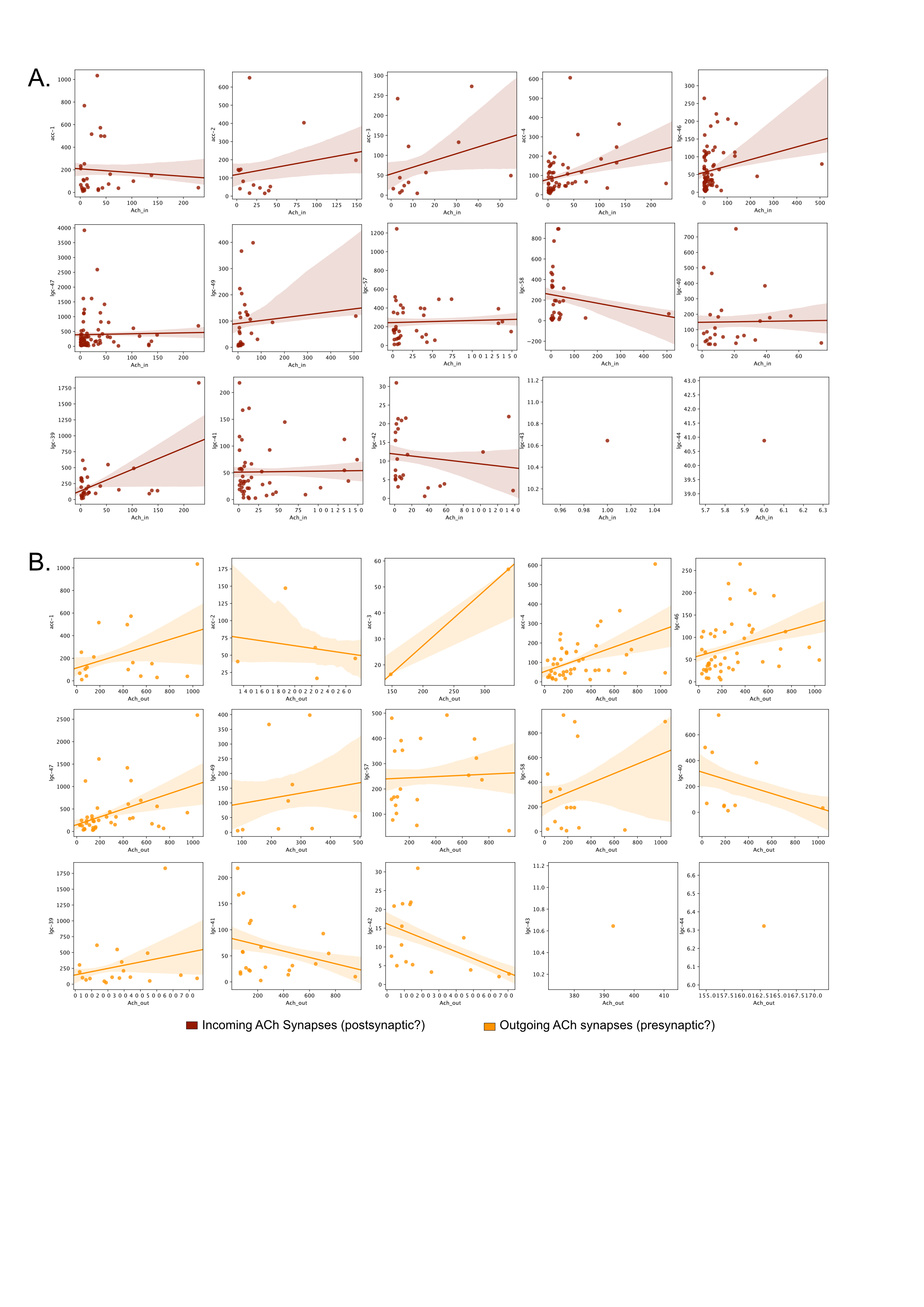

Supplement: Figure 6-1 — Correlation graphs between cholinergic synapses and expression of selected LGICs. Scatter plots showing gene expression level versus the total number of incoming cholinergic synapses (red) and outgoing cholinergic synapses (orange). Lines fit using relplot with shaded areas representing the SE of the line fit. Download Figure 6-1, TIF file. [file ns-JN-RM-1516-22-s16.tif]
