# Supplementary material for: A Novel and Functionally Diverse Class of Acetylcholine-Gated Ion Channels
Source: J Neurosci. 2023 Feb 15;43(7):1111–24. doi: 10.1523/JNEUROSCI.1516-22.2022 (PMC9962794; doi:10.1523/JNEUROSCI.1516-22.2022)
Supplement: Table 4-1 — List of C. elegans strains used in this study. Download Table 4-1, DOCX file. [file ns-JN-RM-1516-22-s02.docx]

Table 4-1: **List of *C. elegans* strains used in this study**

| Strain No. | Description |
| --- | --- |
| AQ4728 | him-5; ljEx1388[lgc-57p::lgc-57::SL2GFP; punc-122:RFP] |
| PHX3594 | lgc-40(syb3594) |
| PHX3536 | lgc-57(syb3536) |
| PHX3562 | lgc-58(syb3562) |
| AQ4614 | ljEx1332[lgc-39p(2kb)::lgc-39::SL2 GFP; punc-122::RFP] |
| AQ4427 | ljEx1254[lgc-40p(2kb)::mKate2::gpd-23'UTR; unc-122::gfp] |
| AQ4423 | ljEx1252[lgc-57p(2kb)::mKate2::gpd-23'UTR; unc-122::gfp] |
| AQ4377 | ljEx1242[lgc-39p(2kb)::mKate2::gpd-23'UTR; unc-122::gfp] |
| AQ4428 | ljEx1255[lgc-58p(2kb)::mKate2::gpd-23'UTR; unc-122::gfp] |
| AQ5072 | ljEx1562[lgc-46p::GFP::gpd-2UTR; punc-122::RFP] |
| OH15262 | “NeuroPAL” otEx7057 |
| AQ4657 | lgc-39(lj121) |
| AQ4808 | NeuroPal(otEx7057); IjEx1438[lgc-42p::lgc-42::SL2 GFP; unc-122::RFP] |
| AQ4852 | NeuroPal(otEx7057); IjEx1453 [lgc-43p::lgc-43::SL2 GFP; unc-122::RFP] |
| AQ4529 | him-5(e1490); IjEx1299[lgc-48p::gDNA lgc-48 3'UTR::SL2 mKate2;punc-122::GFP] |
| AQ4928 | IjEx1498 [lgc-47p(2kb)::lgc-47::SL2 GFP; unc-122::RFP] |
| AQ4849 | NeuroPal(otEx7057); IjEx1453 [lgc-49p::lgc-49::SL2 GFP; unc-122::RFP] |
| AQ4927 | IjEx1497[lgc-45ap::lgc-45a::SL2 GFP; unc-122::RFP] |

Note:

*lgc-57* previously called *ggr-1*

*lgc-58* previously called *ggr-2*
