# Supplementary material for: A Novel and Functionally Diverse Class of Acetylcholine-Gated Ion Channels
Source: J Neurosci. 2023 Feb 15;43(7):1111–24. doi: 10.1523/JNEUROSCI.1516-22.2022 (PMC9962794; doi:10.1523/JNEUROSCI.1516-22.2022)
Supplement: Table 4-2 — List of primers used in this study. Download Table 4-2, DOCX file. [file ns-JN-RM-1516-22-s03.docx]

Table 4-2: **List of primers used in this study**

| **Description** | **Sequence** |
| --- | --- |
| ggr-1 gene F | GGGGACAAGTTTGTACAAAAAAGCAGGCTTAatgcattccttattcctgaaaattctgatatacag |
| ggr-1 gene R | GGGGACCACTTTGTACAAGAAAGCTGGGTTttaaagcaaaagttcaggggaattgct |
| ggr-2 gene F | GGGGACAAGTTTGTACAAAAAAGCAGGCTTAatgcttcttgtctcttcaatattcttattactgtacag |
| ggr-2 gene F | GGGGACCACTTTGTACAAGAAAGCTGGGTTatgggatgtgaaatatgaaaaattatacacttggtt |
| ggr-2 promoter F | GGGGACAACTTTGTATAGAAAAGTTGTAccccggaaagcatctggtaaac |
| ggr-2 promoter R | GGGGACTGCTTTTTTGTACAAACTTGTaatgccgtcgtggtaagacgttatagttgac |
| lgc-39 promoter F | GGGGACAACTTTGTATAGAAAAGTTGTAatcgttttgtgggacacctttagg |
| lgc-39 promoter R | GGGGACTGCTTTTTTGTACAAACTTGTcgatgattcacatcagggatgcttcaac |
| lgc40_gene_F | GGGGACAAGTTTGTACAAAAAAGCAGGCTTAatcgaaactatgagccaactaaaatccaaa |
| lgc40_gene_R | GGGGACCACTTTGTACAAGAAAGCTGGGTTatacataataatttattttcaaattaaaatggctaatatcatcatcgt |
| plgc40_promoter_F | GGGGACAACTTTGTATAGAAAAGTTGTAatacgggtcattcttgccct |
| plgc40_promoter_R | GGGGACTGCTTTTTTGTACAAACTTGTctgaaattccaaaatctggcgtcaattagattga |
| lgc45c_P221_F | GGGGACAAGTTTGTACAAAAAAGCAGGCTTAatggactacaatctatcgactgatgactttgaggagt |
| lgc45ac_P221_R | GGGGACCACTTTGTACAAGAAAGCTGGGTTgacaagacagccacaaaaaaataacaacg |
| lgc45a_P221_F | GGGGACAAGTTTGTACAAAAAAGCAGGCTTAatgccacgtcataaccatttcatcc |
| plgc45c_P4P1r_F | GGGGACAACTTTGTATAGAAAAGTTGTAaacacgcttagcggaccaaa |
| plgc45c_P4P1r_R | GGGGACTGCTTTTTTGTACAAACTTGTggccttgtagcttgtgtcaatccataaaac |
| plgc45a_P4P1r_F | GGGGACAACTTTGTATAGAAAAGTTGTAgttttccgcccatcccca |
| plgc45a_P4P1r_R | GGGGACTGCTTTTTTGTACAAACTTGTtttgtgacgggtgcatctggaatt |
| plgc43short_P4P1r_F | GGGGACAACTTTGTATAGAAAAGTTGTAattgaatggttttaaactaaacacaaaagcctg |
| plgc43short_P4P1r_R | GGGGACTGCTTTTTTGTACAAACTTGTttttcatccattttttaggtcatagattgtagaatatagtagagg |
| lgc44_P221_F | GGGGACAAGTTTGTACAAAAAAGCAGGCTTAatgtctcttcttatctctatttctcttctttttctaatatttcc |
| lgc44_P221_R | GGGGACCACTTTGTACAAGAAAGCTGGGTTgttcatacacatttattcaatttttaatacactttctctcatctcc |
| plgc44_P4P1r_F | GGGGACAACTTTGTATAGAAAAGTTGTAagcgaattggagcttttgct |
| plgc44_P4P1r_R | GGGGACTGCTTTTTTGTACAAACTTGTcgattgatgtcttctacattgaaatcagaaatagagt |
| lgc42_P221_F | GGGGACAAGTTTGTACAAAAAAGCAGGCTTAatgacttctaatcgatggcttctattttttttattgt |
| lgc42_P221_R | GGGGACCACTTTGTACAAGAAAGCTGGGTTtcagtccattaatgggggttctcgt |
| plgc-42_P4P1r_F_v2 | GGGGACAACTTTGTATAGAAAAGTTGTAggcgtgtcctggaaaatgtgt |
| plgc-42_P4P1r_R_v2 | GGGGACTGCTTTTTTGTACAAACTTGTtttaatccaaaacgtcaactcaaaacgggg |
| gRNA1_lgc39_sens | TTGgtaattgcgaataataccga |
| gRNA1_lgc39_anti | AACtcggtattattcgcaattac |
| lgc39_3hom_F | GGCTGCTCTTCgACGggatccgacctgaatcgg |
| lgc39_3hom_R | GGGTGCTCTTCgTACacgacctttttggaagttaatgga |
| lgc39_5hom_F | GGCTGCTCTTCgTGGtgagattttggaagcaatttcc |
| lgc39_5hom_R_noPAM | GGGTGCTCTTCgCATttcggtattattcgcaattactgg |
| ggr2_GFP_F | gtaccggcggtagtgGAAGAAGTTTGGGCAGAACAAAAAGAAAC |
| ggr2_GFP_R | aacaattcttctcctttacTCATTTGGGCCTCAGCGTCTT |
| ggr1_GFP_F | gtaccggcggtagtgGTGGCGCAGTACAGGGAG |
| ggr1_GFP_R | aacaattcttctcctttacTCATCATGTCATGTACTTGCTCCGAGAG |
| lgc40_GFP_F | gtaccggcggtagtgGGAGCGGCCCGAGATATAGAC |
| lgc40_GFP_R | aacaattcttctcctttacTCATGAACAATGTTGAGAGAGCAGTGTCG |
| lgc39_GFP_KSM_F | ggcggtagtGGATCCGACCTGAATCGGAATAAAAAATCTCAGCA |
| lgc39_GFP_KSM_R | ctttacTCATCTCGGTATTATTCGCAATTACTGGCTTGTTG |
| lgc46 w promo SL2GFP_f | GAAACAGCTATGACgtactacagtacgccaagcttt |
| lgc46 w promo SL2GFP_r | taggatgagacagcaaattctgagcaaagttcaaaaagtgg |
| plgc-46_SL2_part1_r | tccaactaaactctttccctca |
| plgc-46_SL2_part2_f | gagtttagttggaaatttggccac |
| lgc47a promo SL2GFP_f | AAACAGCTATGACcttccccacatttgatctcga |
| lgc47a promo SL2GFP_part1_R | ATTCCCTGCTGATTAATTGCTC |
| lgc47a promo SL2GFP_part2_F | TCAGCAGGGAATCATGAAATGT |
| lgc47a w promo SL2GFP_r | gtaggatgagacagcgcatacacgtgtgacataattgaatatg |
| lgc-48_p221_F | GGGGACAAGTTTGTACAAAAAAGCAGGCTTAATGTTTTTTCATATTTTTTTGGGCCTACTGG |
| lgc-48_p221_R | GGGGACCACTTTGTACAAGAAAGCTGGGTTTTATCCAACTTTGAAACCATATCGGGC |
| lgc-48_P4P1r_F | GGGGACAACTTTGTATAGAAAAGTTGTAaattcaaagaaactgatgcaacgaatgg |
| lgc-48_P4P1r_R | GGGGACTGCTTTTTTGTACAAACTTGTgaagctgctgaataaaatgaaaaagaatgagc |
| lgc49 promo SL2GFP_f | GAAACAGCTATGACcgttttgattagtttttaatcggcaca |
| lgc49 promo SL2GFP_r | taggatgagacagccacaatttacaaaaactgggacgatg |
