# Supplementary material for: A Novel and Functionally Diverse Class of Acetylcholine-Gated Ion Channels
Source: J Neurosci. 2023 Feb 15;43(7):1111–24. doi: 10.1523/JNEUROSCI.1516-22.2022 (PMC9962794; doi:10.1523/JNEUROSCI.1516-22.2022)
Supplement: Table 5-1 — The list shows ligand identity and ion selectivity for LGICs in C. elegans. Each gene is assigned a polarity based upon its ion selectivity; this is shown in column Pos/Neg as follows: P, positive (cation selectivity); or N, negative (anion selectivity). For orphan receptors, the ligand and ion selectivity have been predicted based on homology; this has been noted in the column Inferred as yes. The references where the receptor has a validated ligand are from experiments evaluating receptors using electrophysiological characterization in a heterologous expression system. Download Table 5-1, DOCX file. [file ns-JN-RM-1516-22-s05.docx]

Table 5-1: **The list shows ligand identity and ion selectivity for LGICs in *C. elegans*.** Each gene is assigned a polarity based upon its ion selectivity, this is shown in column ‘Pos/Neg’ as P: positive (cation selectivity) or N: negative (anion selectivity). For orphan receptors the ligand and ion selectivity has been predicted based on homology, this has been noted in the column ‘Inferred’ as ‘yes’. The references where the receptor has a validated ligand are from experiments evaluating receptors using electrophysiological characterisation in a heterologous expression system.

| **Gene** | **Gated** | **Pos/Neg** | **Inferred?** | **References for heterologous characterisation** |
| --- | --- | --- | --- | --- |
| *acr-2* | ACh | P |  | (Jospin et al., 2009) |
| *acr-3* | ACh | P |  | (Jospin et al., 2009) |
| *acr-6* | ACh | P | yes |  |
| *acr-7* | ACh | P | yes |  |
| *acr-8* | ACh | P | yes |  |
| *acr-9* | ACh | P | yes |  |
| *acr-10* | ACh | P | yes |  |
| *acr-11* | ACh | P | yes |  |
| *acr-12* | ACh | P |  | (Petrash et al., 2013) |
| *lev-8* | ACh | P |  | (Towers et al., 2005) |
| *acr-14* | ACh | P | yes |  |
| *acr-15* | ACh | P | yes |  |
| *acr-16* | ACh | P |  | (Touroutine et al., 2005) |
| *acr-19* | ACh | P | yes |  |
| *acr-25* | ACh | P | yes |  |
| *eat-2* | ACh | P |  | (McKay et al., 2004) |
| *lev-1* | ACh | P |  | (Fleming et al., 1997) |
| *unc-29* | ACh | P |  | (Fleming et al., 1997) |
| *unc-38* | ACh | P |  | (Fleming et al., 1997) |
| *unc-63* | ACh | P |  | (Culetto et al., 2004) |
| *unc-49* | GABA | N |  | (Bamber et al., 1999) |
| *exp-1* | GABA | P |  | (Beg and Jorgensen, 2003) |
| *lgc-35* | GABA | P |  | (Jobson et al., 2015) |
| *lgc-36* | GABA | P | yes |  |
| *lgc-37* | GABA | N | yes |  |
| *lgc-38* | GABA | N | yes |  |
| *avr-14* | Glu | N |  | (Keane and Avery, 2003) |
| *avr-15* | Glu | N |  | (Keane and Avery, 2003) |
| *glc-1* | Glu | N |  | (Dent et al., 2000) |
| *glc-2* | Glu | N |  | (Cully et al., 1994) |
| *glc-3* | Glu | N |  | (Horoszok et al., 2001) |
| *glc-4* | Glu | N | yes |  |
| *acc-2* | ACh | N |  | (Putrenko et al., 2005) |
| *acc-3* | ACh | N |  | (Putrenko et al., 2005) |
| *acc-4* | ACh | P | yes |  |
| *lgc-46* | ACh | N |  | (Takayanagi-Kiya et al., 2016; Liu et al., 2017) |
| *lgc-47* | ACh | N | yes |  |
| *lgc-48* | ACh | P | yes |  |
| *lgc-49* | ACh | N |  | This study |
| *ggr-1/lgc-57* | choline, ACh | N |  | This study |
| *ggr-2/lgc-58* | choline, ACh | N |  | This study |
| *lgc-39* | ACh, tyramine, octopamine | N |  | This study |
| *lgc-40* | choline, ACh | N |  | This study |
| *gab-1* | GABA | N |  | (Feng et al., 2002) |
| *acc-1* | ACh | N |  | (Putrenko et al., 2005) |
| *nmr-1* | Glu | P |  | (Brockie et al., 2001) |
| *nmr-2* | Glu | P |  | (Kano et al., 2008) |
| *glr-1* | Glu | P |  | (Maricq et al., 1995; Rose et al., 2003; Walker et al., 2006) |
| *glr-2* | Glu | P |  | (Maricq et al., 1995; Rose et al., 2003; Walker et al., 2006) |
| *glr-3* | Glu | P | Yes |  |
| *glr-4* | Glu | P | Yes |  |
| *glr-5* | Glu | P |  | (Zou et al., 2018) |
| *glr-6* | Glu | P | yes |  |
| *glr-7* | Glu | P | yes |  |
| *glr-8* | Glu | P | yes |  |

**References:**

Bamber BA, Beg AA, Twyman RE, Jorgensen EM (1999) The Caenorhabditis elegans unc-49 locus encodes multiple subunits of a heteromultimeric GABA receptor. J Neurosci 19.

Beg AA, Jorgensen EM (2003) EXP-1 is an excitatory GABA-gated cation channel. Nat Neurosci 6.

Brockie PJ, Mellem JE, Hills T, Madsen DM, Maricq A V. (2001) The C. elegans glutamate receptor subunit NMR-1 is required for slow NMDA-activated currents that regulate reversal frequency during locomotion. Neuron 31.

Culetto E, Baylis HA, Richmond JE, Jones AK, Fleming JT, Squire MD, Lewis JA, Sattelle DB (2004) The Caenorhabditis elegans unc-63 gene encodes a levamisole-sensitive nicotinic acetylcholine receptor α subunit. J Biol Chem 279.

Cully DF, Vassilatis DK, Liu KK, Paress PS, Van Der Ploeg LHT, Schaeffer JM, Arena JP (1994) Cloning of an avermectin-sensitive glutamate-gated chloride channel from Caenorhabditis elegans. Nature.

Dent JA, Smith MM, Vassilatis DK, Avery L (2000) The genetics of ivermectin resistance in Caenorhabditis elegans. Proc Natl Acad Sci U S A 97.

Feng XP, Hayashi J, Beech RN, Prichard RK (2002) Study of the nematode putative GABA type-A receptor subunits: Evidence for modulation by ivermectin. J Neurochem 83.

Fleming JT, Squire MD, Barnes TM, Tornoe C, Matsuda K, Ahnn J, Fire A, Sulston JE, Barnard EA, Sattelle DB, Lewis JA (1997) Caenorhabditis elegans levamisole resistance genes lev-1, unc-29, and unc-38 encode functional nicotinic acetylcholine receptor subunits. J Neurosci 17.

Horoszok L, Raymond V, Sattelle DB, Wolstenholme AJ (2001) GLC-3: A novel fipronil and BIDN-sensitive, but picrotoxinin-insensitive, L-glutamate-gated chloride channel subunit from Caenorhabditis elegans. Br J Pharmacol 132.

Jobson MA, Valdez CM, Gardner J, Garcia LR, Jorgensen EM, Beg AA (2015) Spillover transmission is mediated by the excitatory GABA receptor LGC-35 in C. elegans. J Neurosci.

Jospin M, Qi YB, Stawicki TM, Boulin T, Schuske KR, Horvitz HR, Bessereau JL, Jorgensen EM, Jin Y (2009) A neuronal acetylcholine receptor regulates the balance of muscle excitation and inhibition in Caenorhabditis elegans. PLoS Biol 7.

Kano T, Brockie PJ, Sassa T, Fujimoto H, Kawahara Y, Iino Y, Mellem JE, Madsen DM, Hosono R, Maricq A V. (2008) Memory in Caenorhabditis elegans Is Mediated by NMDA-Type Ionotropic Glutamate Receptors. Curr Biol 18.

Keane J, Avery L (2003) Mechanosensory inputs influence Caenorhabditis elegans pharyngeal activity via ivermectin sensitivity genes. Genetics 164.

Liu P, Chen B, Mailler R, Wang ZW (2017) Antidromic-rectifying gap junctions amplify chemical transmission at functionally mixed electrical-chemical synapses. Nat Commun.

Maricq A V., Peckol E, Driscoll M, Bargmann CI (1995) Mechanosensory signalling in C. Elegans mediated by the GLR-1 glutamate receptor. Nature 378.

McKay JP, Raizen DM, Gottschalk A, Schafer WR, Avery L (2004) eat-2 and eat-18 are Required for Nicotinic Neurotransmission in the Caenorhabditis elegans Pharynx. Genetics 166.

Petrash HA, Philbrook A, Haburcak M, Barbagallo B, Francis MM (2013) ACR-12 ionotropic acetylcholine receptor complexes regulate inhibitory motor neuron activity in Caenorhabditis elegans. J Neurosci 33.

Putrenko I, Zakikhani M, Dent JA (2005) A family of acetylcholine-gated chloride channel subunits in Caenorhabditis elegans. J Biol Chem 280.

Rose JK, Kaun KR, Chen SH, Rankin CH (2003) GLR-1, a Non-NMDA Glutamate Receptor Homolog, Is Critical for Long-Term Memory in Caenorhabditis elegans. J Neurosci 23.

Takayanagi-Kiya S, Zhou K, Jin Y (2016) Release-dependent feedback inhibition by a presynaptically localized ligand-gated anion channel. Elife.

Touroutine D, Fox RM, Von Stetina SE, Burdina A, Miller DM, Richmond JE (2005) acr-16 encodes an essential subunit of the levamisole-resistant nicotinic receptor at the Caenorhabditis elegans neuromuscular junction. J Biol Chem 280.

Towers PR, Edwards B, Richmond JE, Sattelle DB (2005) The Caenorhabditis elegans lev-8 gene encodes a novel type of nicotinic acetylcholine receptor α subunit. J Neurochem 93.

Walker CS, Francis MM, Brockie PJ, Madsen DM, Zheng Y, Maricq A V. (2006) Conserved SOL-1 proteins regulate ionotropic glutamate receptor desensitization. Proc Natl Acad Sci U S A 103.

Zou W, Fu J, Zhang H, Du K, Huang W, Yu J, Li S, Fan Y, Baylis HA, Gao S, Xiao R, Ji W, Kang L, Xu T (2018) Decoding the intensity of sensory input by two glutamate receptors in one C. elegans interneuron. Nat Commun 9.
